# Secondary Organic Aerosol Formation during the Oxidation of Large Aromatic and Other Cyclic Anthropogenic Volatile Organic Compounds

**DOI:** 10.1021/acsestair.4c00176

**Published:** 2024-10-03

**Authors:** Damianos Pavlidis, Petro Uruci, Kalliopi Florou, Andrea Simonati, Christina Ν. Vasilakopoulou, Georgia Argyropoulou, Spyros N. Pandis

**Affiliations:** † Department of Chemical Engineering, University of Patras, Patras, GR 26504, Greece; ‡ Institute of Chemical Engineering Sciences (FORTH/ICE-HT), Patras, GR 26504, Greece

**Keywords:** SOA, VOCs, IVOCs, aromatics, cyclohexanes, high NO_*x*_, VBS parametrization

## Abstract

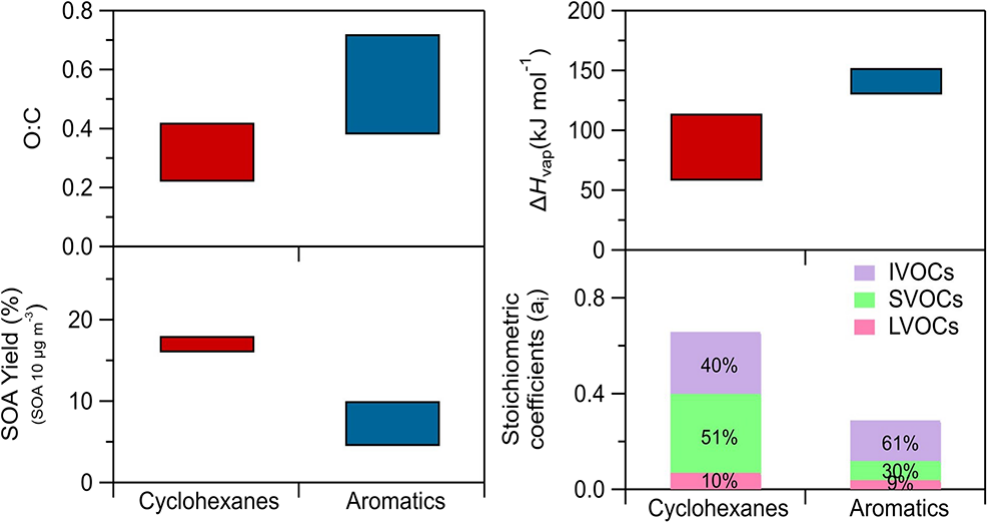

The secondary organic aerosol (SOA) production from the reactions of anthropogenic large volatile (VOCs) and intermediate volatility organic compounds (IVOCs) with hydroxyl radicals under high NO_*x*_ conditions was investigated. The organic compounds studied include cyclic alkanes of increasing size (amylcyclohexane, hexylcyclohexane, nonylcyclohexane, and decylcyclohexane) and aromatic compounds (1,3,5-trimethylbenzene, 1,3,5-triethylbenzene and 1,3,5-tri*tert*-butylbenzene). A considerable amount of SOA was formed from all examined compounds. For the studied cyclohexanes (C_11_–C_16_) there appears that the SOA yield depends nonlinearly on the length of their substitute chain. The large cyclohexanes had higher yields than the aromatic compounds, but the aromatic precursors produced a more oxidized SOA. This was due to the production of lower volatility and O:C first generation products by the cyclohexanes. Most oxidation products (with C* < 10^4^ μg m^–3^) in the case of cyclohexanes are SVOCs (∼50%), while of aromatics are IVOCs (∼60%). Structure, molecular size, and length of the substitute chain of the parent hydrocarbon were found to play key roles in SOA formation, oxidation state, and volatility. The SOA volatility distribution, effective vaporization enthalpy, and effective accommodation coefficient were also quantified by combining SOA yields, thermodenuder (TD) and isothermal dilution measurements. Parameterizations for the Volatility Basis Set (VBS) are proposed for future use in chemical transport models.

## Abstract 2

SOA production from large anthropogenic aromatic and cyclic alkanes oxidized with hydroxyl radicals under high NO_*x*_ conditions is explored and parameterized for the Volatility Basis Set.

## 1 Introduction

Volatile (VOCs, having an effective saturation concentration C* greater than 10^6^ μg m^–3^), intermediate volatility (IVOCs, C* between 10^3^–10^6^ μg m^–3^), and semivolatile organic compounds (SVOCs, C* between 10^–1^–10^3^ μg m^–3^) are emitted into the atmosphere from biogenic and anthropogenic sources. These organic vapors can undergo atmospheric oxidation, forming a secondary organic aerosol (SOA), as their low volatility oxidation products condense in the particulate phase. In most environments, SOA is the dominant component of the organic aerosol (OA), and its presence has significant impacts on human health, visibility, and the Earth’s climate.^1,2^ Several studies have indicated that IVOCs may have an important role in atmospheric SOA formation.^2−7^ These molecules have lower initial volatility and also lower emissions than the traditional SOA precursors (light aromatics, isoprene, monoterpenes, etc.), which have received most of the scientific attention during the last decades. However, they are expected to have much higher SOA yields than smaller VOCs.^2,5,6,8^ Current atmospheric chemical transport models (CTMs) often do not include these vapors and tend to underpredict the measured SOA levels, especially in urban areas.^4,7,9^ Αn important reason behind this, is the lack of experimental data for SOA production by IVOCs.^10^

Most of the work until now has focused on the ability of VOCs with 5 to 10 carbon atoms to produce SOA in the atmosphere. Highly studied categories of such hydrocarbons are aromatics,^11,12^ monoterpenes,^13,14^ cycloalkenes^8,15^ and aliphatic alkanes.^8,16−19^ Two larger VOC that have received some attention are 1,3,5-trimethylbenzene and its isomers^20−25^ and hexylcyclohexane^26,27^ with most studies focusing on their oxidation mechanisms rather than SOA formation and properties (e.g., volatility, vaporization enthalpy, hygroscopicity, etc.).

The volatility of organic compounds determines to a large extent their partitioning between the gas and particulate phases.^28,29^ Donahue et al.^30^ and Stanier et al.^31^ developed the volatility basis set (VBS) to simulate the formation and evolution of SOA in chemical transport models (CTMs) based on the volatility distribution of OA. Estimating the volatility distribution of SOA in laboratory experiments can be a challenge.^32^ Uruci et al.^33^ suggested that the SOA formation parameterization for the VBS is improved by combining yield, thermodenuder (TD) and dilution measurements.

The main goal of this work is to study the SOA production from the reactions of large individual anthropogenic VOCs and IVOCs with hydroxyl radicals (OH), under high NO_*x*_ conditions (above 200 ppb), often encountered in urban areas. More specifically, the study aims to quantify the SOA yields of each VOC precursor as a function of the SOA levels at room temperature and to estimate the volatility distribution of their oxidation products using the algorithm of Uruci et al.^33^ The effects of the structure of the compound (alkylic cycle and aromatic ring) and the size of the molecule on the SOA yields are also investigated. The ultimate objective and contribution of this research is to provide results that can be used in CTMs, to improve our understanding of the contribution of IVOCs to SOA formation in urban areas.

The organic compounds that were studied include cyclic alkanes of increasing size (amylcyclohexane, hexylcyclohexane, nonylcyclohexane, and decylcyclohexane) and also aromatic compounds (1,3,5-trimethylbenzene, 1,3,5-triethylbenzene, and 1,3,5-tri*tert*-butylbenzene). Table 1 lists the compounds studied along with their structure, chemical formula, and characterization according to their volatility (Table S1).

**Table 1:**
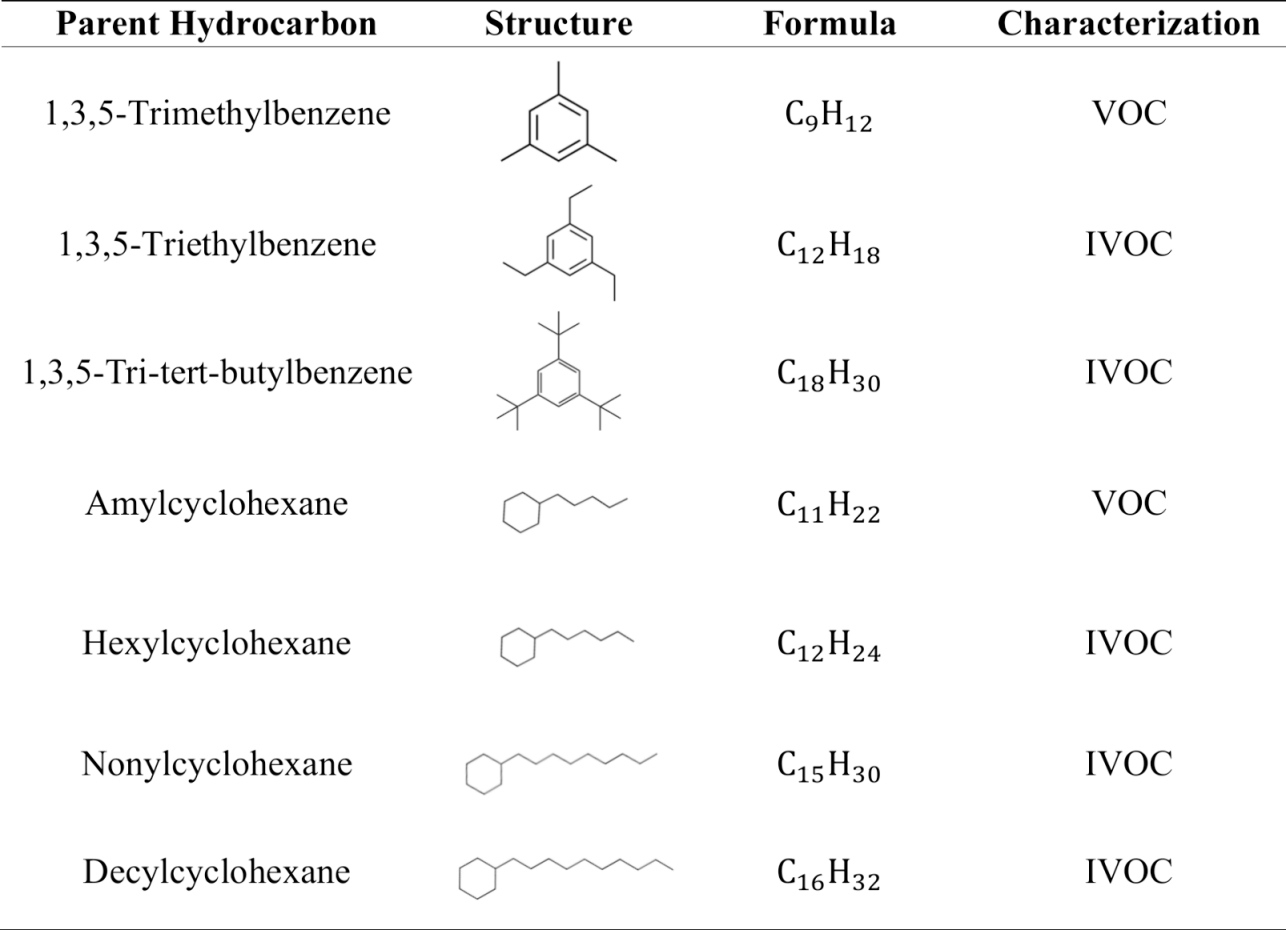
Organic Compounds Studied

## 2 Methodology

### 2.1 Instrumentation and Experimental Procedure

Photo-oxidation experiments were carried out in the atmospheric simulation chamber of the Foundation for Research and Technology-Hellas (FORTH-ASC). The chamber is a 10 m^3^ Teflon reactor located inside a 30 m^3^ room. The walls of the room are covered with aluminum mirrors and UV lights (Osram, L36W/73; *J*_NO_2__ = 0.59 h^–1^) to increase the homogeneity and intensity of light inside the reactor, and the temperature of the room is controlled.

**Figure 1:**
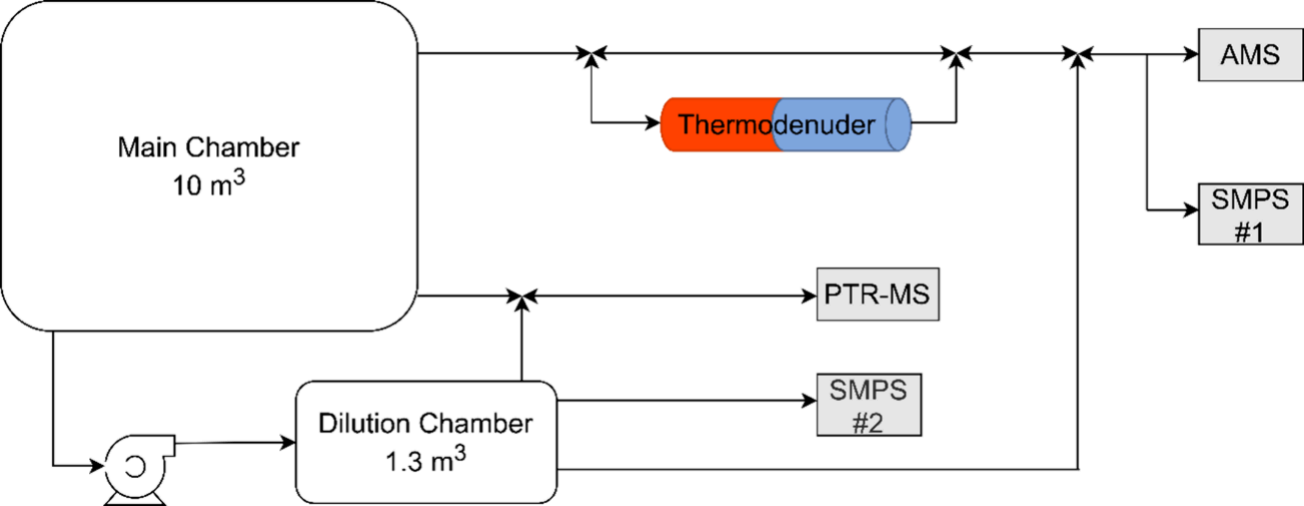
Schematic of the experimental setup.

The experimental setup is depicted in Figure 1. A scanning mobility particle sizer (SMPS) that consists of a classifier (SMPS#1; TSI model 3080), a differential mobility analyzer (DMA; TSI model 3081), and a condensation particle counter (CPC; TSI model 3775) was used to measure the particle size distributions in the 20–980 nm size range. A high-resolution time-of-flight aerosol mass spectrometer (HR-ToF-AMS) was utilized to measure the mass concentration and composition of the aerosol. The concentration of VOCs was monitored by a Proton-Transfer-Reaction-Mass-Spectrometer (PTR-QMS 500, Ionicon Analytik). Measurements of the nitrogen oxide (NO_*x*_) and ozone (O_3_) concentrations were obtained from gas monitors (Teledyne models: T201, 400E, respectively). The temperature and relative humidity of the chamber were measured using an Omega sensor (RH-USB).

The chamber was filled before each experiment with ambient compressed air that passed through a series of filters removing particles (high-efficiency particulate air filter, HEPA), gas-phase organics (activated-carbon filters), NO_*x*_ (Purafil filter), and water vapor (silica gel). Particles can be lost to the chamber walls and their loss rate is greatly affected by static charges. To reduce this an air ionizer (Dr. Schneider SL-001) was used prior to each experiment.

Following the reactor filling process, a solution of 1 g L^–1^ ammonium sulfate in ultrapure water was introduced into an atomizer (model 3076, TSI) passing through a silica gel dryer to produce dry ammonium sulfate particles. The seed aerosol provides a surface for the SOA components to condense on reducing artifacts due to condensation on the walls. Isotopically labeled butanol (1-butanol-*d*_9_) was injected through a septum orifice to the chamber to track the OH concentration. Butanol-*d*_9_ was selected as an OH tracer based on the work of Barmet et al.^34^ These authors determined that butanol-*d*_9_ was the most suitable among several tested compounds. For the next 2 h, the reactor remained unperturbed to characterize the particle losses on its walls. After the VOC precursor was introduced, nitrous acid (HONO, 33.3 mM) was added into the chamber via a bubbler. Finally, the UV lights were turned on and SOA formation started as the precursor compounds reacted with the OH radicals produced by the rapid photolysis of HONO. Because 1,3,5-tri*tert*-butylbenzene is solid at room temperature, a custom-made flash vaporizer was used to introduce it to the chamber (Figure S1).

Between experiments, the reactor underwent a thorough cleaning process. Initially, it was flushed with dry, clean air for at least 12 h. In an effort to remove any lingering material adhering to the chamber walls, HONO was introduced into the chamber, and the UV lights were turned on for 1.5 h, followed by an ozone injection and another 1.5 h of photo-oxidation. In the final cleaning step, clean air was introduced again in the chamber, passing through it for at least 12 h.

One experiment for each of the studied compounds was conducted in order to characterize the SOA volatility distribution by thermodenude (TD) and isothermal dilution measurements. Details for the design of the TD used can be found in Louvaris et al.^35^ The temperature of the TD ranged approximately from 25 to 170 °C and was increasing stepwise every 15 min. The TD was heated until 90% of the SOA evaporated or its concentration reached a plateau. The aerosol is transferred to the dilution chamber to be isothermally diluted. Isothermal dilution is found to be essential for characterizing the aerosol’s volatility as a constant temperature is maintained while the aerosol concentration is reduced to atmospheric levels. This approach improves the accuracy of the volatility distribution estimation, as its results are not dependent on the enthalpy of evaporation. The AMS and SMPS switched sampling between the main chamber (bypass) and the TD every 4 min by using computer-controlled valves. Thermodenuder measurements started as soon as the reactions were completed (minimum of 1 h). The residence time at the centerline of the heating section of the TD was 106 s at room temperature.

A metal bellows pump (model MB 602) was used to transfer the formed SOA to the dilution chamber (Teflon reactor, 1.3 m^3^) after the reactions were completed (minimum 1 h). The dilution chamber was already partially filled with clean dry air. The pump operated with a flow of 80 L min^–1^ and was used for 2.5 min. Before using the dilution chamber, purified air was passed through it for at least 2 days. The distribution of the transferred aerosol was continuously monitored by an additional SMPS (SMPS#2; TSI, classifier model 3080, DMA model 3081, and CPC model 3787). Moreover, the AMS and SMPS #1 alternated sampling between the main chamber and the dilution chamber every 16 min by a three-way valve with measurements taken over 8 min intervals. The PTR-MS switched every 40 min and measured for 4 min the gas phase in the dilution chamber. At the end of the experiment, the dilution chamber was flushed for about an hour (i.e., until the particle mass concentration was less than 1 μg m^–3^), was filled with purified air and ammonium sulfate particles were added. The chamber was then left undisturbed for 1.5 h to characterize the particle wall losses.

### 2.2 Data Analysis

#### 2.2.1 AMS

The HR data analysis in this work was performed using the ToF-AMS analysis software SQUIRREL v1.57Ι and the HR-ToF-AMS data analysis software Peak Integration by Key Analysis (PIKA v1.16Ι) adapted in Igor Pro 6 (Wavemetrics). The AMS measures the nonrefractory components (organics, nitrate, sulfate, ammonium, and chloride) of the sampled aerosol. To correct the AMS measurements, a size dependent collection efficiency (CE) is estimated and applied in each experiment, following Pavlidis et al.^36^ The average CE estimated and used is 0.77 ± 0.07.

#### 2.2.2 Particle Wall-Loss Correction

In each experiment, a size-dependent wall-loss rate constant, corrected for coagulation (*k*(*D*_p_)) was calculated following the approach of Nah et al.^37^ by utilizing the evolution of the particle size distribution measured by the SMPS from the seeds-only period of the experiment.

For the correction of the AMS measurements, we took advantage of the relatively flat profile of *k*(*D*_p_) in the AMS range and used a size independent constant by averaging *k*(*D*_p_) in that range. In our study, the size independent particle wall-loss rate constant ranged from 0.01 to 0.1 h^–1^.

#### 2.2.3 Dilution Ratio

The dilution ratio in each experiment was determined as the concentration of 1-butanol-*d*_9_ measured by PTR-MS (mass-to-charge ratio, *m*/*z* 66) in the dilution chamber divided by its concentration in the main chamber.

#### 2.2.4 TD Loss Correction

The measurements obtained by sampling from the TD were corrected using a size- and temperature-dependent particle loss constant (Figure S2), as particles can be lost to the walls of the TD. At 25 °C, 3% of the particles are lost, with losses increasing to 30% at 200 °C. The loss constants were experimentally characterized according to Cain and Pandis.^38^ Sodium chloride particles were introduced into the smog chamber and sequentially passed through the bypass and the TD operating at different temperatures. The particle losses were quantified by comparing the SMPS distributions in the bypass and TD, for every size bin and temperature.

#### 2.2.5 Pump Loss Correction

The aerosol measurements of the dilution chamber were also corrected for particle losses that occurred within the transfer system between the two chambers. The size-dependent transfer losses (Figure S3) were quantified by comparing the size distribution of ammonium sulfate particles in principal to that in the dilution chamber, accounting for the dilution ratio. For the particle sizes measured in this study, 40% ± 18% of the particles were lost during the transfer process. Loss corrections for the VOCs were not applied as for the particular pump, Kaltsonoudis et al.^39^ reported that VOC losses are negligible. Losses of IVOCs were not quantified in that study due to limitations of the PTR-MS method used.

#### 2.2.6 SOA Yield

To quantify SOA formation in each experiment, the fractional aerosol mass yield (*Y*_SOA_), defined as the fraction of the reacted VOC that has been converted to aerosol, was estimated based on
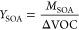
1where *M*_SOA_ is the SOA mass concentration and ΔVOC is the mass concentration of the VOC that has reacted during the photo-oxidation. In this study, the first-generation SOA yields are estimated as the initial oxidation of the precursor is investigated. The formed SOA mass concentration is calculated as the average of the maximum values obtained from the SMPS and CE corrected AMS during the reaction period after the applied wall-loss corrections to their measurements. The SOA mass concentration from the SMPS measurements is obtained by subtracting the volume concentration of ammonium sulfate from the total volume concentration and multiplying this difference with the SOA density estimated by the algorithm of Kostenidou et al.^40^ The sum of organics, nitrate, and water is considered as the AMS-based SOA for these experiments. At the low relative humidity of the experiments, practically all the AMS water is the result of the fragmentation of organic molecules. Overall, the AMS-based SOA has a mean deviation of 4% from the SMPS-based SOA. The concentrations of all of these components started to increase once the reactions began. The practical absence of gas-phase ammonia (measured by Teledyne model, T201) indicates that organonitrates are formed, while due to low RH conditions (lower than 13%), water vapor cannot contribute significantly to the particle mass. In our experiments, for ammonium nitrate formation to occur, the concentration of ammonia would need to be several ppb. Therefore, practically all of the detected nitrate concentration corresponds to organonitrates, and the detected water by the AMS is produced mainly by the fragmentation of organic molecules.

Only two out of the seven precursors were detected by PTR-MS, the 1,3,5-trimethylbenzene (*m*/*z* 121) and 1,3,5-triethylbenzene (*m*/*z* 163). This is because the instrument has unit mass resolution and can accurately detect VOCs with *m*/*z* values lower than 165. For the rest of the compounds, their initial gas concentration levels were determined based on the injected mass to the main chamber. Based on the OH levels in the chamber and the reaction constants, we can assume that more than 95% of the precursors reacted during our experiments.

#### 2.2.7 Particle Mass Fraction Remaining

Thermograms, plots of particle mass fraction remaining (MFRs) as a function of TD temperature, were generated by dividing the TD SOA mass concentration by the bypass (BP) SOA mass concentration. The thermograms of each experiment represent the average MFRs derived from the SMPS and AMS measurements, given that the corresponding measurements were quite consistent with each other. To calculate the SOA mass concentration in the TD from the SMPS, the seed mass concentration was subtracted from the total concentration (Figure S4).

Areograms, MFRs in the dilution chamber as a function of dilution time (“areo” is dilute in Greek), were generated using the SOA mass concentration obtained from the second SMPS divided by the initial mass concentration in the dilution chamber. The SOA mass concentration in the dilution chamber was estimated by multiplying the total volume with the organic aerosol density obtained by the Kostenidou et al.^40^ algorithm after subtracting the ammonium sulfate volume based on the AMS measurements. Similar to Cain et al.^32^ rapid evaporation of the SOA was observed in some experiments. To account for this, the initial SOA concentration of the dilution chamber was determined by the SOA concentration of the main chamber right before the transfer began, correcting for pump transfer losses.

#### 2.2.8 SOA Formation Parameters for the VBS

The SOA volatility distributions of the studied oxidation systems were estimated using the algorithm of Uruci et al.^33^ This algorithm uses yield, TD and isothermal dilution measurements as inputs to estimate the volatility distribution of the SOA, the vaporization enthalpy, the mass accommodation coefficient (*a*_m_) and their uncertainties. The algorithm also estimates the yields and their uncertainties for different atmospheric conditions (temperature and SOA levels).

In this algorithm, the SOA formation is based on the assumption of the formation of a pseudoideal organic solution in the particulate phase in equilibrium with the gas phase, as described by Donahue et al.^30^ and Strader et al.^41^ The effective saturation concentrations at various temperatures are calculated using the Clausius–Clapeyron equation. Time-dependent evaporation in the TD and dilution chamber is modeled using the dynamic mass transfer model proposed by Riipinen et al.,^42^ assuming a monodisperse aerosol population.

The domain of the parameters was discretized, and then all combinations of volatilities, the effective vaporization enthalpy (Δ*H*_vap_), and the accommodation coefficient were estimated. In this work, we used five volatility bins with stoichiometric coefficients varying from 0.0 to 0.8, with values of 0.0, 0.05, 0.1, 0.15, 0.2, 0.3, 0.4, 0.6, and 0.8. The values used for Δ*H*_vap_ were from 20 to 200 kJ mol^–1^ with a stepwise increase of 20 kJ mol^–1^ and, for *a*_m_, the values were 0.001, 0.01, 0.1, and 1. Approximately 620000 simulations were performed, from which we identified those that led to a normalized mean square error of less than 5%. Then we calculated the best estimate following the approach described in Uruci et al.^33^

## 3 Results and Discussion

In total, 31 experiments were conducted, and at least 3 experiments for each compound were studied. A summary of the results of each experiment is presented in Table 2, while information about temperature and relative humidity can be found in Table S2.

**Table 2:** Initial Conditions and Results of the Experiments

exp. No.	seeds^a^ (μg m^–3^)	[ΟΗ] × 10^7^ (molecules cm^–3^)	Δ[VOC]^b^ (ppb)	NO_*x*_^c^(ppb)	ρ_SOA_ (g cm^–3^)	*M*_SOA_^d^ (μg m^–3^)	SOA yield (%)
Amylcyclohexane							
AC1	29.2	3.6	7	–e	1.30 ± 0.32	5.1 ± 0.6	11.4 ± 1.4
AC2	35.6	4.0	14	–	1.31 ± 0.06	15.8 ± 0.4	17.5 ± 0.4
AC3	31.7	4.2	28	–	1.28 ± 0.01	44.4 ± 0.3	24.5 ± 0.2
AC4	51.3	4.3	54	–	1.20 ± 0.01	117.1 ± 0.5	34.1 ± 0.1
Hexylcyclohexane							
HC1	28.1	3.9	13	–	1.38 ± 0.10	16.9 ± 0.7	18.5 ± 0.7
HC2	33.9	4.5	20	760	1.31 ± 0.05	32.8 ± 0.7	23.9 ± 0.5
HC3	28.5	3.8	26	–	1.30 ± 0.03	44.3 ± 0.6	24.2 ± 0.3
HC4	38.6	3.8	52	600	1.31 ± 0.06	123.1 ± 2.6	33.6 ± 0.7
HC5	33.4	4.6	–f	–	1.22 ± 0.03	105.4 ± 1.3	—
Nonylcyclohexane							
NC1	19.5	5.4	5	570	1.59 ± 0.45	3.6 ± 0.6	7.7 ± 1.3
NC2	19.7	5.5	11	520	1.24 ± 0.09	19.0 ± 0.8	20.5 ± 0.8
NC3	21.5	4.1	16	615	1.28 ± 0.06	32.2 ± 0.7	23.1 ± 0.5
NC4	19.3	4.0	32	455	1.05 ± 0.03	95.8 ± 1.1	34.5 ± 0.4
NC5	26.0	6.6	32	685	1.14 ± 0.03	126.9 ± 1.7	45.7 ± 0.6
Decylcyclohexane							
DC1	39.6	4.9	19	640	1.30 ± 0.06	19.6 ± 0.5	10.9 ± 0.3
DC2	58.4	3.3	24	–	1.49 ± 0.03	37.4 ± 0.4	16.6 ± 0.2
DC3	41.0	3.9	29	645	1.28 ± 0.04	44.3 ± 0.7	16.3 ± 0.3
DC4	41.1	4.8	39	645	1.15 ± 0.01	69.0 ± 0.4	19.1 ± 0.1
DC5	42.7	4.9	39	570	1.10 ± 0.02	95.9 ± 0.9	26.5 ± 0.3
DC6	66.9	5.0	58	295	1.26 ± 0.01	226.1 ± 1.1	41.7 ± 0.2
1,3,5-Trimethylbenzene							
TMB1	27.2	1.5	101	–	1.61 ± 0.48	5.5 ± 0.8	1.1 ± 0.2
TMB2	33.9	3.2	210	–	1.42 ± 0.03	34.2 ± 0.4	3.3 ± 0.0
TMB3	35.2	3.3	395	790	1.43 ± 0.07	135.4 ± 3.3	6.9 ± 0.2
1,3,5-Triethylbenzene							
TEB1^g^	27.7	1.6	31	–	1.53 ± 0.36	3.7 ± 0.4	1.8 ± 0.2
TEB2	35.6	1.9	113	–	1.55 ± 0.14	15.1 ± 0.7	2.0 ± 0.1
TEB3	32.8	1.7	241	–	1.35 ± 0.02	43.8 ± 0.3	2.7 ± 0.0
TEB4	36.1	3.1	449	965	1.33 ± 0.06	239.6 ± 5.1	7.9 ± 0.2
1,3,5-Tri*tert*-butylbenzene							
TTB1	32.7	3.8	27	690	1.43 ± 0.08	8.4 ± 0.3	3.0 ± 0.1
TTB2	30.6	5.5	33	–	1.45 ± 0.06	26.5 ± 0.6	7.9 ± 0.2
TTB3	32.6	4.5	60	1110	1.20 ± 0.04	117.2 ± 1.9	19.2 ± 0.3
TTB4	41.1	4.3	81	1220	1.19 ± 0.04	186.8 ± 3.5	22.4 ± 0.4

a Seeds mass concentration based on the raw SMPS measurement before the lights were on (*t* = 0 h), using a density of 1.77 g cm^–3^.

b VOC concentration of 1,3,5-trimethylbenzene and 1,3,5-triethylbenzene was obtained from PTR-MS, for 1,3,5-tri*tert*-butylbenzene and for cyclohexanes was calculated based on the injection volume assuming that all the VOC reacted.

c Average initial concentration of NO_*x*_.

d SOA mass concentration as an average of HR-ToF-AMS and SMPS.

e The symbol “–“ corresponds to no data.

f Unknown injection volume.

g 66% of UV lights were on.

The SOA concentrations formed ranged from approximately 4 μg of m^–3^ to almost 300 μg of m^–3^. For the volatility characterization experiments, the SOA concentration levels were close to or above 100 μg m^–3^. This allowed us to include compounds with higher volatility by enhancing their partitioning into the particle phase compared to the atmospheric conditions, under which they might exist solely as vapors. Additional information and results for the experiments (AC4, HC5, NC4, DC6, TMB3, TEB4, and TTB3) used to characterize the volatility distribution of SOA can be found in Table S3 and Figures S5–S11, respectively. The estimated VBS parameters are listed in Table 3.

**Table 3:** Estimated VBS Parameters^a^

Δ*H*_vap_ (kJ mol^–1^)	log(*a*_m_)	stoichiometric coefficients (*a*_*i*_)				
		10^–1^	10^0^	10^1^	10^2^	10^3^
Amylcyclohexane						
113.8	–0.86	0.066	0.047	0.079	0.171	0.283
±29.4	±0.72	±0.037	±0.045	±0.06	±0.09	±0.199
Hexylcyclohexane						
79.3	–0.85	0.067	0.059	0.092	0.144	0.27
±21.7	±0.73	±0.042	±0.052	±0.064	±0.085	±0.195
Nonylcyclohexane						
57.7	–0.84	0.066	0.030	0.074	0.377	0.197
±17.5	±0.70	±0.027	±0.034	±0.064	±0.123	±0.156
Decylcyclohexane						
65.7	–0.61	0.047	0.051	0.086	0.120	0.276
±11.0	±0.49	±0.034	±0.045	±0.059	±0.069	±0.195
1,3,5-Trimethylbenzene						
151.5	–0.39	0.020	0.029	0.001	0.044	0.168
±35.2	±0.54	±0.025	±0.025	±0.008	±0.035	±0.139
1,3,5-Triethylbenzene						
130.4	–1.23	0.010	0.030	0.010	0.026	0.135
±35.7	±0.99	±0.020	±0.025	±0.020	±0.029	±0.082
1,3,5-Tri*tert*-butylbenzene						
149.8	–1.25	0.049	0.016	0.068	0.024	0.191
±30.7	±0.82	±0.008	±0.023	±0.037	±0.041	±0.139

a The uncertainty in the estimates (±σ) is also included.

In all experiments the average OH concentration was a little above 10^7^ molecules cm^–3^, the average temperature was 20.6 ± 0.6 °C, the average RH (%) 12.5 ± 0.4 and the average NO_*x*_ was 690 ± 110 ppb.

### 3.1 Cyclohexanes

#### 3.1.1 SOA Mass Yields

**Figure 2:**
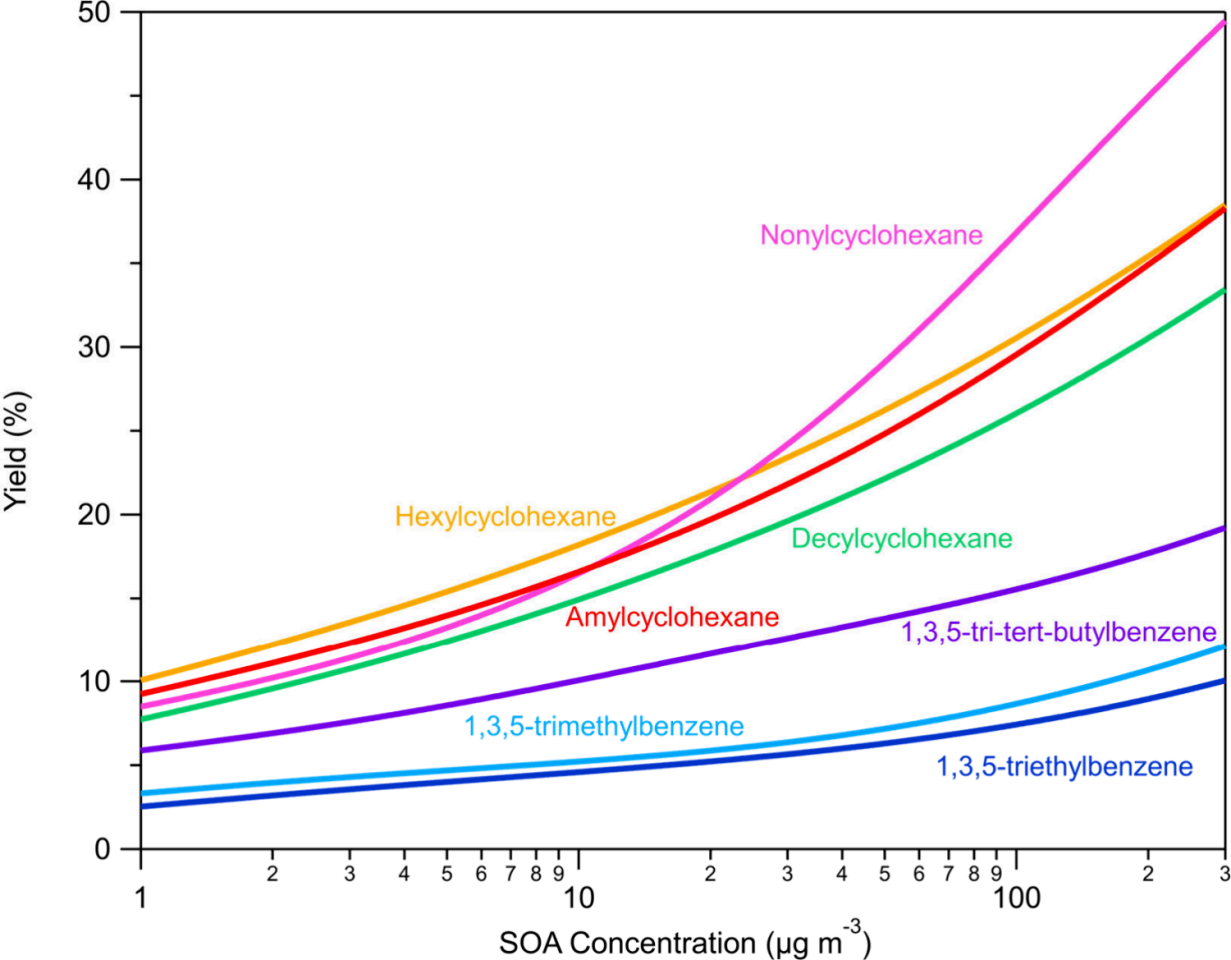
SOA yield fits for the examined VOCs as a function of aerosol mass produced. The yield fits are estimated by the Uruci et al.^33^ algorithm. The corresponding data were corrected for particle wall losses.

The first-generation SOA yields of the studied parent hydrocarbons are listed in Figure 2. First-generation SOA forms shortly after the initial oxidation begins, whereas later-generation SOA results from further reactions of the initial oxidation products. In our experiments, we focus solely on the initial oxidation (first-generation) and do not proceed to the aging (second-generation) of these products, which would require a second injection of HONO to generate additional OH radicals for further reactions. The fitting algorithm of Uruci et al.^33^ fits with the same VBS parameters the yield measurements along with the TD and isothermal dilution measurements. As a result, this approach leads to greater apparent discrepancies between the yield measurements and the VBS fits, as shown in Figures S5c–11c, compared to fitting only the yield data. These results suggest that the SOA yield depends nonlinearly on the length of the chain and the overall size of the molecule for these studied cyclohexanes (11–16 carbon atoms). The yields of the various cyclohexanes were quite similar in the OA range below 20 μg m^–3^. At higher SOA levels, nonylcyclohexane (C_15_H_30_) presented higher yields than did amylcyclohexane (C_11_H_22_) and hexylcyclohexane (C_12_H_24_). Schilling et al.^27^ found an SOA yield of 42% at 975 μg m^–3^ of OA for the oxidation of hexylcyclohexane which is consistent with our value of 48%. On the other hand the amylcyclohexane SOA yield at 15 μg m^–3^ measured in our study is significantly higher than the one reported by Tkacik et al.^2^ (18% in this study versus 5% in the previous work under similar experimental conditions). The reasons for this discrepancy are not clear.

#### 3.1.2 SOA Composition

**Figure 3:**
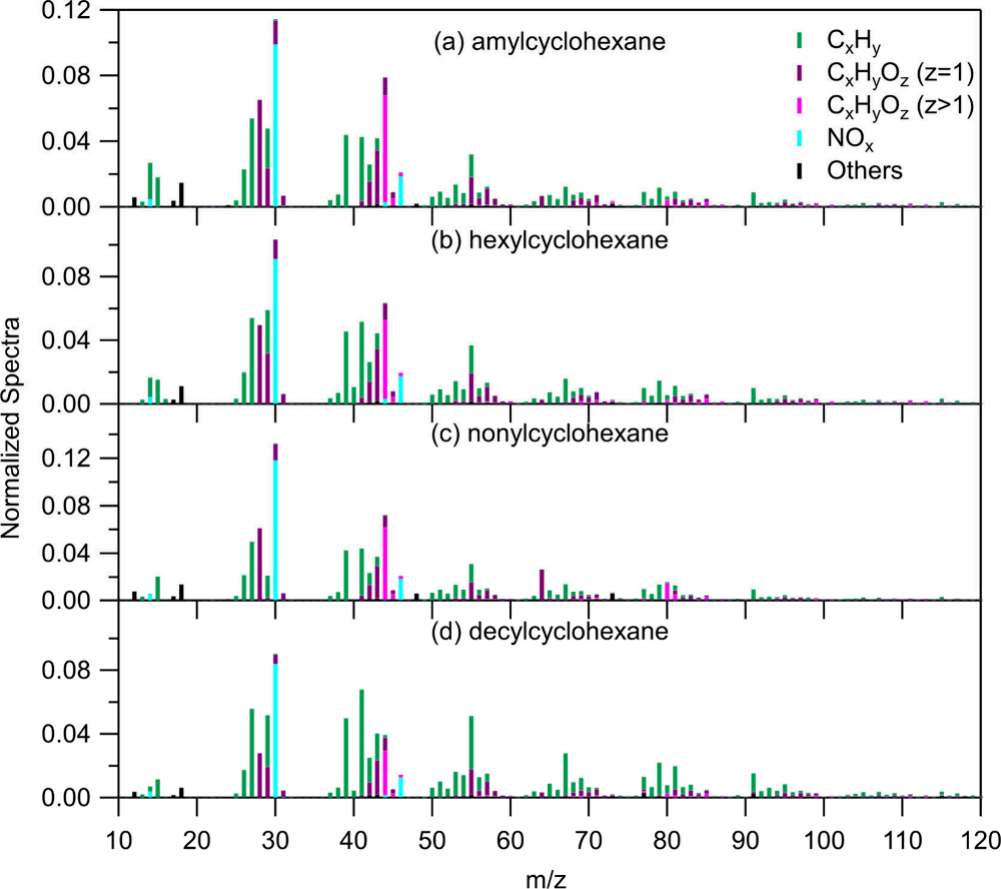
Normalized HR AMS spectra of the SOA formed from the oxidation of (a) amylcyclohexane, (b) hexylcyclohexane, (c) nonylcyclohexane (NC1), and (d) decylcyclohexane. Water is excluded.

The AMS mass spectra of the SOA produced during the oxidation of each cyclohexane are shown in Figure 3.

If the high-resolution AMS spectra are treated as vectors in the corresponding space, the angle between them is defined as their theta angle.^43^ Two AMS spectra are considered similar enough if their theta angle is below 15°. The highest theta angle among the experiments of amylcyclohexane was 12° (between AC1 and AC4), of hexylcyclohexane 7° (between HC1 and HC4), of nonylcyclohexane 8° (between NC2 and NC4) and of decylcyclohexane 14° (between DC1 and DC6), indicating similar composition between the experiments. Experiment NC1 has a theta angle ranging from 17° to 26° to the other nonylcyclohexane experiments, and the normalized AMS spectrum of its SOA is shown in Figure 3c. These high theta angles are considered to occur because of the low SOA mass concentrations formed in the NC1 experiment which are closer to ambient levels and thus its AMS mass spectrum obtained is more representative of real-world conditions. In low concentrations, the role of LVOCs is more significant to the SOA composition compared to higher concentrations where SVOCs and IVOCs dominate the SOA. The average normalized AMS mass spectra of the remaining nonylcyclohexane experiments are shown in Figure S13.

Peaks with strong signals of all the cyclohexanes SOA mass spectra appear at *m*/*z* 27 (C_2_H_3_^+^), 28 (CO^+^), 29 (CHO^+^) (except NC1), 30 (organonitrates), 39 (C_3_H_3_^+^), 41 (C_3_H_5_^+^), 43 (C_2_H_3_O^+^ and C_3_H_7_^+^), 44 (CO_2_^+^), and 55 (C_3_H_3_O^+^ and C_4_H_7_^+^). Other prominent peaks in the SOA mass spectra of all cyclohexanes, which are stronger in the case of decylcyclohexane, exist at *m*/*z* 67, 79, 81, and 91. When comparing Figures 3c and S13, a significant distinction between experiment NC1 and the other nonylcyclohexane experiments is the presence of a strong signal for *m*/*z* 64 (C_4_O^+^) that is exclusive to NC1.

**Figure 4:**
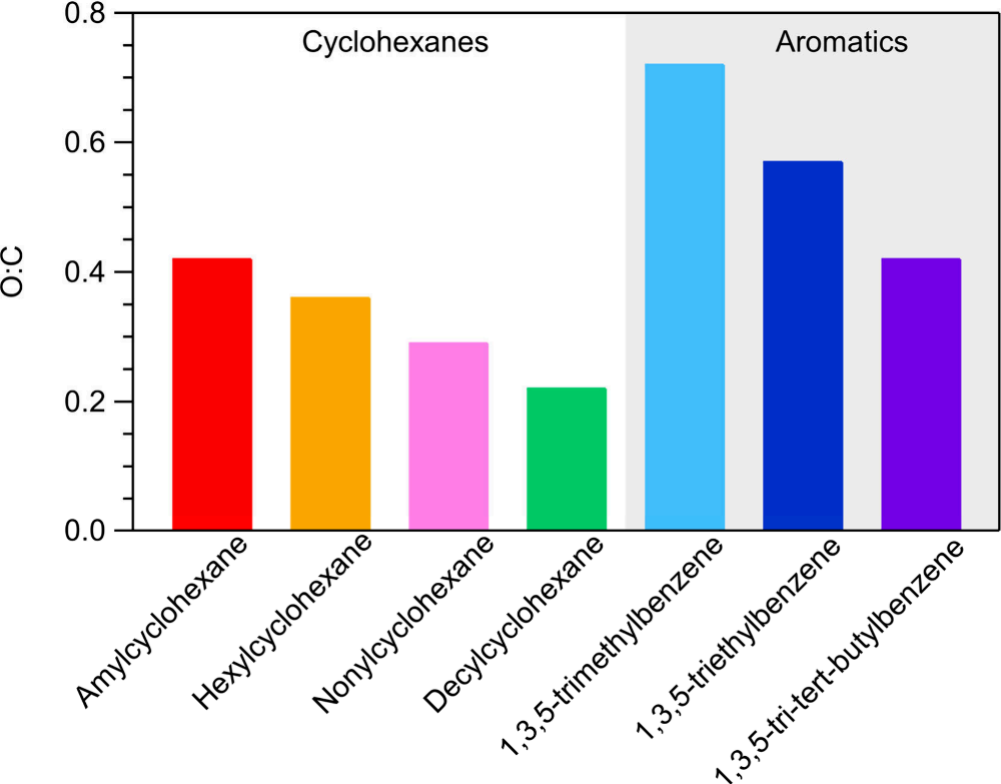
Average O/C ratio of the studied compounds.

The atomic oxygen-to-carbon ratio (O/C) of OA is a useful metric used to characterize the oxidation state of the products that occur from the oxidation of a VOC. In this work the O/C ratio from the AMS measurements is calculated based on the Canagaratna et al.^44^ approach. The most oxidized SOA among the cyclohexanes is produced from the oxidation of amylcyclohexane with an O/C value of 0.42 (Figure 4), while the least oxidized SOA results from decylcyclohexane with an O/C equal to 0.22. The SOA of hexylcyclohexane has an O/C of 0.36, which closely aligns with the values reported by Yee et al.^26^ (O/C = 0.35; low-NO_*x*_ conditions) and Schilling et al.^27^ (O/C = 0.30). As the size of the precursor cyclohexane and thus the length of the substitute chain increase, the SOA O/C ratio decreases. The O/C of the SOA in the NC1 experiment was 0.38, significantly higher than the average of the rest of the nonylcyclohexane experiments that was 0.26. The O/C of the SOA of amylcyclohexane is similar to the one reported in Tkacik et al.,^2^ while the O/C of decylcyclohexane is approximately 0.1 units higher than that in this previous study.

#### 3.1.3 SOA Volatility Distribution

**Figure 5:**
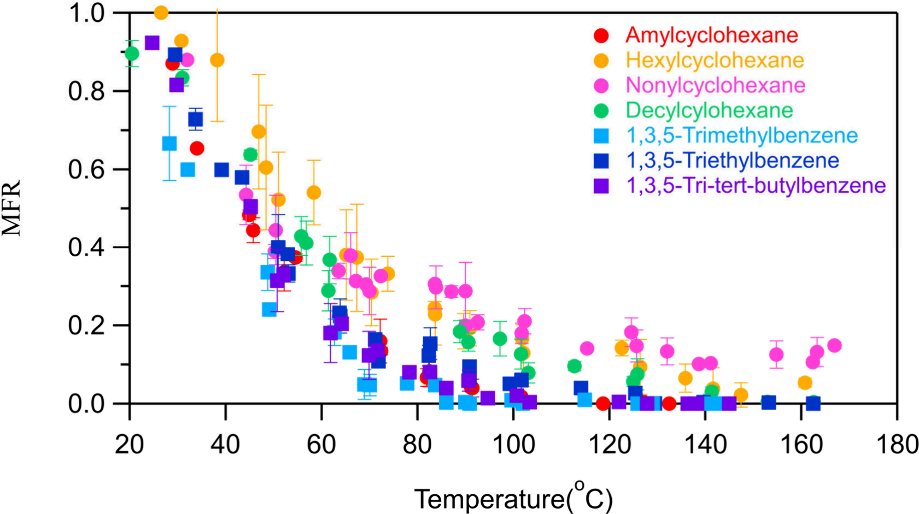
Thermograms of the SOA of each studied precursor. The corresponding experiments were performed with AC4, HC5, NC4, DC6, TMB3, TEB4, and TTB3. The error bars represent the standard deviation of the mean MFR.

The thermograms of all experiments are listed in Figure 5. The SOA from amylcyclohexane evaporates similarly in the TD as the SOA of the three aromatic compounds. Half of the amylcyclohexane’s SOA evaporates around 44 °C, while close to 120 °C it evaporates completely. The initial evaporation of nonylcyclohexane’s SOA closely resembles that of amylcyclohexane, with approximately half of it evaporating at the same temperature. Half of the hexylcyclohexane’s SOA evaporates around 60 °C. Above 140 °C 10–20% of the SOA of hexylcyclohexane and nonylcyclohexane still does not evaporate. Based on these observations, it is expected that the SOA of hexylcyclohexane (C_12_H_24_) and nonylcyclohexane (C_15_H_30_) will have more compounds of lower volatility than the SOA of amylcyclohexane. At approximately 50 °C half of the SOA produced from decylcyclohexane evaporates, while at 160 °C more than 99% of it evaporated.

**Figure 6:**
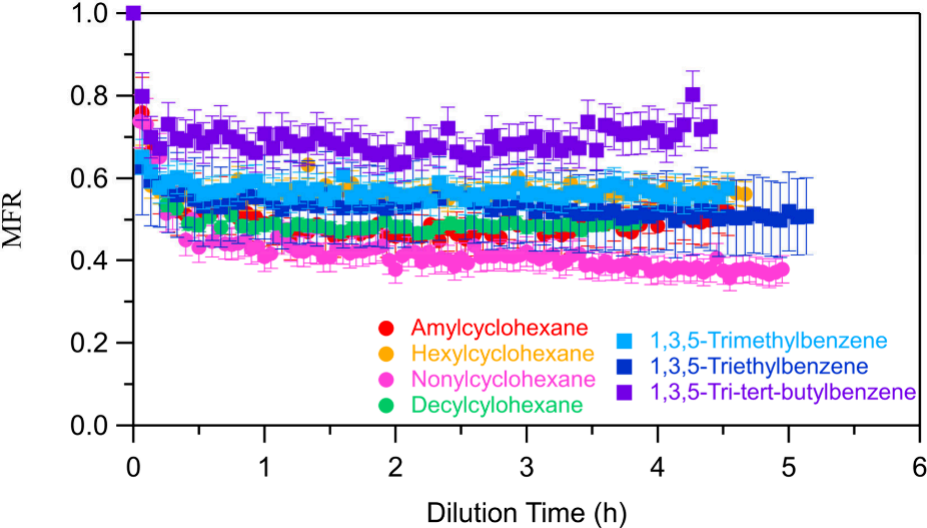
Wall loss-corrected areograms of the SOA of each studied precursor. The error bars represent the corresponding measurement uncertainty.

Figure 6 shows all of the areograms of the SOA isothermal dilution experiments. The SOA of all cyclohexanes reached an equilibrium around 30 min in the dilution chamber. During this first half hour, 50% of amylcyclohexane’s SOA evaporated, along with 45% of hexylcyclohexane’s, 60% of nonylcyclohexane’s, and 52% of decylcyclohexane’s. Among the studied precursors, the SOA of nonylcyclohexane evaporated the most at room temperature, indicating that it contains a significant fraction of relatively volatile compounds.

**Figure 7:**
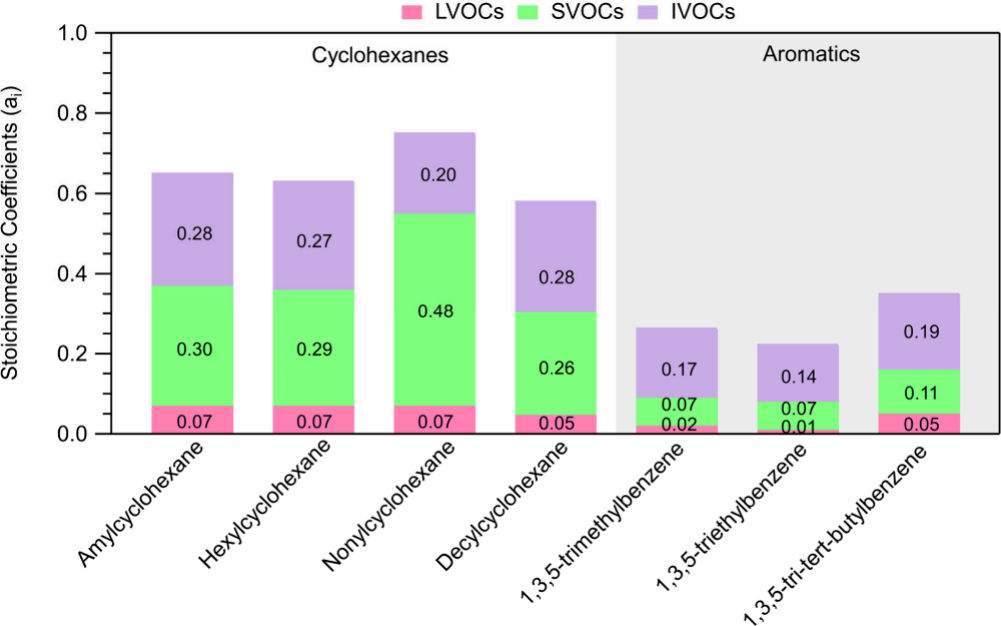
Composition of the oxidation products of each precursor for IVOCs (purple), SVOCs (green), and LVOCs (pink). These are expressed as stoichiometric mass coefficients.

The composition as LVOCs, SVOCs, and IVOCs for the oxidation products of each precursor is summarized in Figure 7. According to these results, the cyclohexanes SOA consisted of around 10% of LVOCs. Except for nonylcyclohexane, around 45% of the oxidation products of the other three cyclohexanes were SVOCs and an additional 45% consisted of IVOCs. The oxidation products in the case of nonylcyclohexane consisted mostly of SVOCs (65%) with only 26% of IVOCs. This higher fraction of SVOC products in the nonylcyclohexane oxidation compared to other cyclohexanes highlights the complex interplay between molecular size and SOA formation, which is influenced by the balance between the formation of high-molecular-weight compounds and fragmentation. Further investigation of the specific chemical pathways involved could provide more insights into this observed behavior. The stoichiometric coefficients of the studied precursors estimated for each volatility bin are depicted in Figure S12.

**Figure 8:**
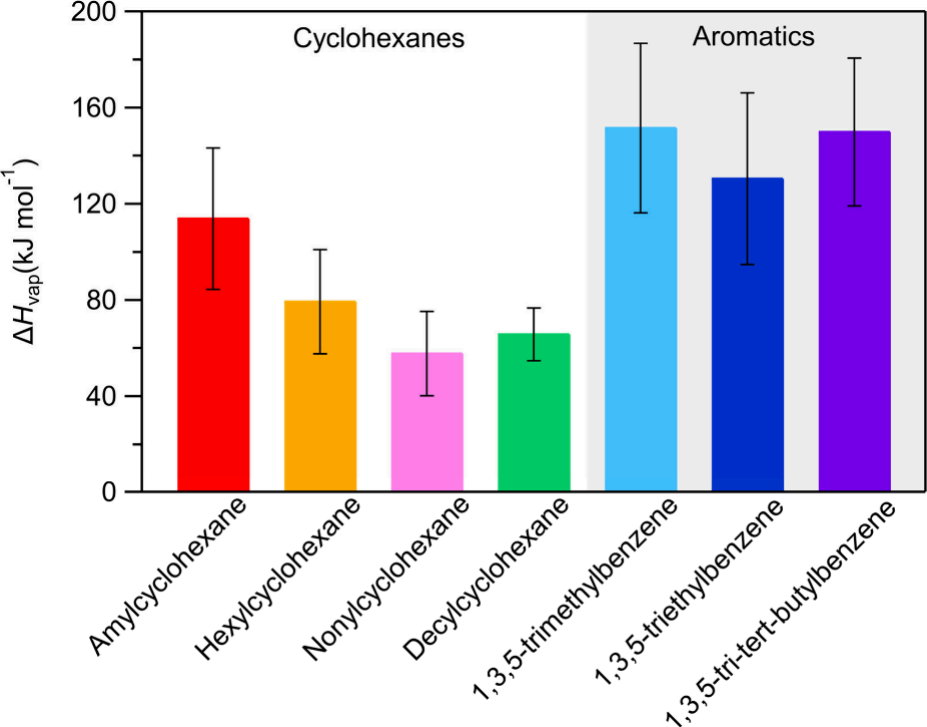
Estimated enthalpy of vaporization of the SOA of each compound oxidized. The error bars represent the uncertainty in the estimated values.

The estimated effective vaporization enthalpies based on the volatility characterization experiments of this study are listed in Figure 8. The SOA of cyclohexanes has a lower effective Δ*H*_vap_ than that of the SOA produced from the aromatics. The SOA from amylcyclohexane approaches the levels of Δ*H*_vap_ of the aromatic compounds’ SOA, with a value of 114 kJ mol^–1^. The rest of cyclohexanes produce SOA with an effective Δ*H*_vap_ around half of the one of the aromatics, with the SOA of hexylcyclohexane having a value of 79 kJ mol^–1^, of nonylcyclohexane 58 kJ mol^–1^ and of decylcyclohexane 66 kJ mol^–1^. Except for decylcyclohexane, as the size of the precursor cyclohexane increases, the estimated effective Δ*H*_vap_ of the SOA decreases. The average uncertainty in the estimated Δ*H*_vap_ of cyclohexanes is 12%.

The SOA of all cyclohexanes, except decylcyclohexane, exhibit a similar value of *a*_m_ around 0.15. The *a*_m_ of the SOA of decylcyclohexane is slightly higher than that of the other cyclohexanes and equal to 0.25. However, all values of *a*_m_ are estimated with significant uncertainty. This suggests that the value of *a*_m_ does not have a significant effect on the results of the algorithm compared to the other parameters. This observation aligns with the findings of Uruci et al.^33^ and Karnezi et al.^45^

### 3.2 Aromatics

#### 3.2.1 SOA Mass Yields

1,3,5-Trimethylbenzene and 1,3,5-triethylbenzene had lower SOA yields compared to those of the other studied compounds, while 1,3,5-tri*tert*-butylbenzene exhibited the highest SOA yield among the currently studied aromatic compounds (Figure 2). For 10 μg m^–3^ of SOA, the yield of 1,3,5-trimethylbenzene is 5.2%, for 1,3,5-triethylbenzene is 4.6% and for 1,3,5-tri*tert*-butylbenzene is 10.1% at these high NO_*x*_ conditions. Paulsen et al.^46^ found an SOA yield of 11.5% at 130 μg m^–3^ for the oxidation of 1,3,5-trimethylbenzene, which is relatively consistent with a 9.4% yield that is estimated based on our measurements.

#### 3.2.2 SOA Composition

**Figure 9:**
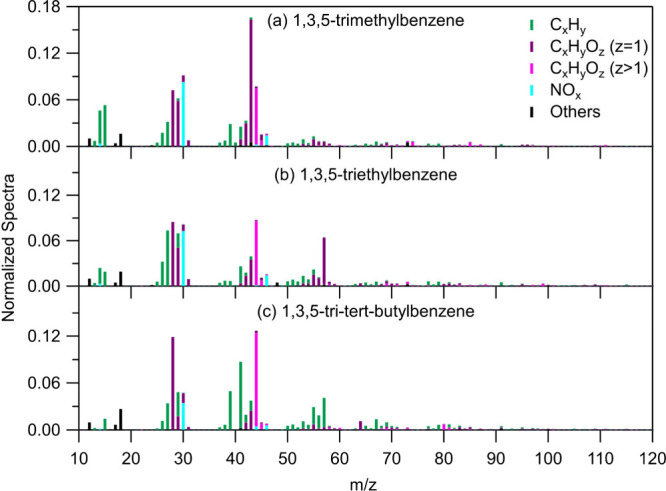
Normalized HR AMS spectra of the SOA formed from the oxidation of (a) 1,3,5-trimethylbenzene, (b) 1,3,5-triethylbenzene, and (c) 1,3,5-tri*tert*-butylbenzene (TTB1). Water is excluded.

The average SOA mass spectrum produced during the oxidation of each aromatic precursor is given in Figure 9. The highest theta angle between the experiments of 1,3,5-trimethylbenzene was found to be 9° (between TMB1 and TMB2), of 1,3,5-triethylbenzene 11° (between TEB1 and TEB4) and of 1,3,5-tri*tert*-butylbenzene 11° (between TTB2 and TTB4), signifying no major changes between the experiments of each compound (good repeatability). For the 1,3,5-tri*tert*-butylbenzene experiments, similar to the case of NC1 experiment, the TTB1 reported high theta angles ranging from 12° to 19° compared to the rest of experiments of 1,3,5-tri*tert*-butylbenzene due to low SOA concentration formed which are closer to ambient levels, and thus, the AMS mass spectrum obtained is more representative of real-world conditions (Figure 9c). The average normalized AMS mass spectrum of the remaining 1,3,5-tri*tert*-butylbenzene experiments is given in Figure S14.

The corresponding SOA spectra had peaks at *m*/*z* 28 (CO^+^), 29 (CHO^+^), 30 (organonitrates), and 44 (CO_2_^+^). *m*/*z* 29, identified as CHO^+^, is consistently present in the SOA mass spectra of all of the studied aromatic compounds, particularly prominent in the SOA of 1,3,5-trimethylbenzene and 1,3,5-triethylbenzene. However, in the case of 1,3,5-tri*tert*-butylbenzene, C_2_H_5_^+^ significantly contributes to *m*/*z* 29. The 1,3,5-trimethylbenzene’s SOA mass spectrum has characteristic peaks at *m*/*z* 14 (CH_2_^+^), 15 (CH_3_^+^), and 43 (C_2_H_3_O^+^). The SOA mass spectra of 1,3,5-triethylbenzene and of 1,3,5-tri*tert*-butylbenzene show a common prominent peak at *m*/*z* 57 corresponding to C_3_H_5_O^+^ and C_4_H_9_^+^, respectively. Also, *m*/*z* 55 (C_4_H_7_^+^) has a notable signal in the SOA mass spectra of 1,3,5-triethylbenzene and of 1,3,5-tri*tert*-butylbenzene. The SOA of 1,3,5-tri*tert*-butylbenzene gives stronger peaks at *m*/*z* 39 (C_3_H_3_^+^) and *m*/*z* 41 (C_3_H_5_^+^) compared to the other aromatics’ SOA while also *m*/*z* 64 (C_4_O^+^) and 67 (C_5_H_7_^+^) are characteristic peaks. The *m*/*z* 27 (C_2_H_3_^+^) appears in all three SOA mass spectra but has a strong signal only for the 1,3,5-triethylbenzene’s SOA.

The most oxidized SOA among the studied compounds is formed from the oxidation of 1,3,5-trimethylbenzene as the average value of the O/C is 0.72 (Figure 4). The least oxidized SOA for the aromatic compounds studied was produced from the oxidation of 1,3,5-tri*tert*-butylbenzene with an O/C ratio of 0.42. However, among the experiments involving 1,3,5-tri*tert*-butylbenzene, TTB1 and TTB2 exhibited an O/C ratio of 0.50, while the other two experiments yielded an O/C of 0.33. The primary disparity between TTB1 and TTB2 compared to TTB3 and TTB4 lies in the levels of SOA, with the latter exceeding 100 μg m^–3^, favoring the partitioning of more volatile oxidation products with lower O/C in the particle phase. A similar observation was discussed by Tkacik et al.^2^ In general, aromatic compounds produce more oxidized SOA compared with the cyclohexanes. Similar to the cyclohexanes, as the size of the aromatic compounds studied and thus the length of their substitute chains increase, the SOA O/C ratio decreases.

#### 3.2.3 SOA Volatility Distribution

The SOA of all three studied aromatic compounds evaporated similarly in the TD (Figure 5). Half of the SOA from both 1,3,5-triethylbenzene and 1,3,5-tri*tert*-butylbenzene evaporated at around 45 °C, whereas the SOA of 1,3,5-trimethylbenzene evaporated approximately at 40 °C. The SOA of 1,3,5-trimethylbenzene evaporated completely roughly at 85 °C, of 1,3,5-tri*tert*-butylbenzene at 100 °C, and of 1,3,5-triethylbenzene at 130 °C.

The areograms of the SOA of the aromatic compounds are given in Figure 6. Equilibrium is reached after 15 min, which is even faster than in the case of cyclohexanes. About 45% of the SOA of 1,3,5-trimethylbenzene and 1,3,5-triethylbenzene evaporated. Approximately 30% of the SOA of 1,3,5-tri*tert*-butylbenzene evaporated, having the lowest evaporation in the dilution chamber among all of the studied precursors. This is an indication that the oxidation products of 1,3,5-tri*tert*-butylbenzene include fewer, more volatile components than the oxidation products of the other two aromatic compounds.

The oxidation of aromatic compounds leads to lower yields (*a*_*i*_) of LVOCs, SVOCs, and IVOCs compared to the cyclohexanes (Figure 7). The oxidation products of the aromatic compounds are more volatile compared to those of cyclohexanes, as approximately 60% of them are IVOCs. For all of the aromatics, around 30% of their oxidation products are SVOCs. For 1,3,5-tri*tert*-butylbenzene, the distributed oxidation products in the LVOC range were almost two times higher than the other two aromatics.

The SOA formed from the oxidation of the aromatic compounds has nearly double the effective Δ*H*_vap_ compared to the one of the cyclohexanes SOA, except the SOA of amylcyclohexane (Figure 8). The SOA of 1,3,5-trimethylbenzene has a Δ*H*_vap_ of 152 kJ mol^–1^, while 1,3,5-tri*tert*-butylbenzene has a similar value of Δ*H*_vap_ equal to 150 kJ mol^–1^ and 1,3,5-triethylbenzene of 130 kJ mol^–1^. The effective Δ*H*_vap_ of the SOA of aromatics is estimated with an average uncertainty of 12%.

The estimated effective accommodation coefficient of the aromatics’ SOA ranged from 0.06 to 0.40 but with significant uncertainty.

These results provide insights into the varying SOA yields and degrees of oxidation exhibited by cyclohexanes and aromatic compounds. The derived parameterizations of this study can be used in atmospheric chemical transport models like PMCAMx-iv^7^ for more accurate simulation of anthropogenic SOA formation related to road transport emissions.

## Data Availability

The data set is publicly accessible at 10.5281/zenodo.10819451. The data of the current study are restricted and can be available from the corresponding author (Spyros N. Pandis) on reasonable request.

## Supporting Information Available

Table with the characteristics of the studied precursors. Table with the experimental conditions. Table with the experimental conditions and properties used in the model for the experiments coupled with TD and isothermal dilution. Figure with the custom-made vaporizer. Figure with the particle loss fraction in the TD as a function of the mobility diameter for different TD temperatures. Figure with the particle losses as a function of mobility diameter during the transfer of aerosol from the main to the dilution chamber. Figure with the thermogram of the ammonium sulfate particles. Figure with the measured and estimated yields of amylcyclohexane. Figure with the measured and estimated yields of hexylcyclohexane. Figure with the measured and estimated yields of nonylcyclohexane. Figure with the measured and estimated yields of decylcyclohexane. Figure with the measured and estimated yields of 1,3,5-trimethylbenzene. Figure with the measured and estimated yields of 1,3,5-triethylbenzene. Figure with the measured and estimated yields of 1,3,5-tri*tert*-butylbenzene. Figure with the estimated volatility distributions of the oxidation products of each precursor. Figure with the average normalized HR AMS spectrum of the SOA formed from the oxidation of nonylcyclohexane. Figure with the average normalized HR AMS spectrum of the SOA formed from the oxidation of 1,3,5-tri*tert*-butylbenzene (PDF)
