# Supplementary material for: Secondary Organic Aerosol Formation during the Oxidation of Large Aromatic and Other Cyclic Anthropogenic Volatile Organic Compounds
Source: ACS EST Air. 2024 Oct 3;1(11):1442–52. doi: 10.1021/acsestair.4c00176 (PMC11555633; doi:10.1021/acsestair.4c00176)
Supplement: Supplementary file 1 — ea4c00176_si_001.pdf [file ea4c00176_si_001.pdf]

Supplementary Material

Secondary organic aerosol formation during the oxidation of large aromatic and other cyclic anthropogenic volatile organic compounds

Damianos Pavlidis[a,b], Petro Uruci[a,b], Kalliopi Florou[b], Andrea Simonati[a,b], Christina N. Vasilakopoulou[a,b], Georgia Argyropoulou[a,b] and Spyros N. Pandis[a,b,*]

[a]Department of Chemical Engineering, University of Patras, Patras, Greece; [b]Institute of Chemical Engineering Sciences (FORTH/ICE-HT), Patras, Greece

Table S1. Characteristics of the studied precursors[1,2].

**Parent Hydrocarbon**	**Molecular weight**	**Saturation Concentration at 298 K** ($\mu g\ m^{-3}$)
1,3,5-Trimethylbenzene	120.2	$1.3x10^7$
1,3,5-Triethylbenzene	162.3	$1.2x10^6$
1,3,5-Tri-tert-butylbenzene	246.4	$8.5x10^6$
Amylcyclohexane	154.3	$4.1x10^6$
Hexylcyclohexane	168.3	$1.7x10^6$
Nonylcyclohexane	210.4	$1.2x10^5$
Decylcyclohexane	224.4	$5.2x10^4$

Table S2. Experimental conditions.

Exp. No.	T (°C)	RH (%)
	**Amylcyclohexane**	
AC1	19.9 ± 0.3	12.6 ± 0.1
AC2	20.0 ± 0.4	12.1 ± 0.1
AC3	20.3 ± 0.3	12.3 ± 0.1
AC4	21.3 ± 0.3	12.0 ± 0.1
	**Hexylcyclohexane**	
HC1	21.1 ± 0.4	12.1 ± 0.1
HC2	19.9 ± 0.3	14.0 ± 0.2
HC3	20.6 ± 0.3	11.5 ± 0.1
HC4	–[a]	–
HC5	21.5 ± 0.3	11.5 ± 0.1
	**Nonylcyclohexane**	
NC1	20.9 ± 0.4	12.6 ± 0.1
NC2	20.0 ± 0.3	12.9 ± 0.1
NC3	19.5 ± 0.3	12.7 ± 0.1
NC4	22.3 ± 0.3	11.8 ± 0.1
NC5	20.4 ± 0.3	13.6 ± 0.1
	**Decylcyclohexane**	
DC1	20.1 ± 0.3	12.8 ± 0.1
DC2	20.4 ± 0.4	11.1 ± 0.1
DC3	20.5 ± 0.3	13.1 ± 0.1
DC4	-	-
DC5	21.5 ± 0.3	13.0 ± 0.1
DC5	21.6 ± 0.4	11.1 ± 0.1
	**1,3,5-trimethylbenzene**	
TMB1	–	–
TMB2	–	–
TMB3	21.1 ± 0.3	11.6 ± 0.1
	**1,3,5-triethylbenzene**	
TEB1	–	–
TEB2	–	–
TEB3	–	–
TEB4	21.4 ± 0.3	12.2 ± 0.1
	**1,3,5-tri-tert-butylbenzene**	
TTB1	19.8 ± 0.3	13.7 ± 0.1
TTB2	20.0 ± 0.4	12.4 ± 0.2
TTB3	21.4 ± 0.2	12.4 ± 0.1
TTB4	20.0 ± 0.3	14.2 ± 0.2

[a] The symbol "–" corresponds to no data.

Table S3. Experimental conditions and properties used in the model for the experiments coupled with TD and isothermal dilution.

Exp. No.	Process	SOA Concentration[a] (μg m^{-3})	Mean Volume Diameter (nm)	SOA Density (g cm^{-3})	Residence Time[b] (s)	Dilution Ratio
AC4	TD	84.5	317.9	1.20	106	—[c]
	Dilution	114.3	318.0	1.28	16,320	9.4
HC5	TD	78.4	311.4	1.22	106	—
	Dilution	102.1	304.5	1.23	17,040	8.1
NC4	TD	61.3	295.0	1.05	106	—
	Dilution	94.3	334.8	1.13	17,820	6.7
DC6	TD	182.6	266.5	1.26	80	—
	Dilution	215.7	262.5	1.27	13,500	9.4
TMB3	TD	71.6	293.6	1.43	106	—
	Dilution	135.4	309.8	1.48	16,320	7.8
TEB4	TD	167.1	320.9	1.33	106	—
	Dilution	239.6	329.8	1.35	18,481	9.7
TTB3	TD	70.8	310.4	1.20	106	—
	Dilution	117.2	313.4	1.25	15,840	9.6

[a] Average SOA concentration in the bypass of the TD and initial SOA concentration of the main chamber right before the transfer to the dilution chamber.
[b] Residence time of the aerosol in the heating tube of the TD and in the dilution chamber after the transfer.
[c] The symbol "–" corresponds to no corresponding data.

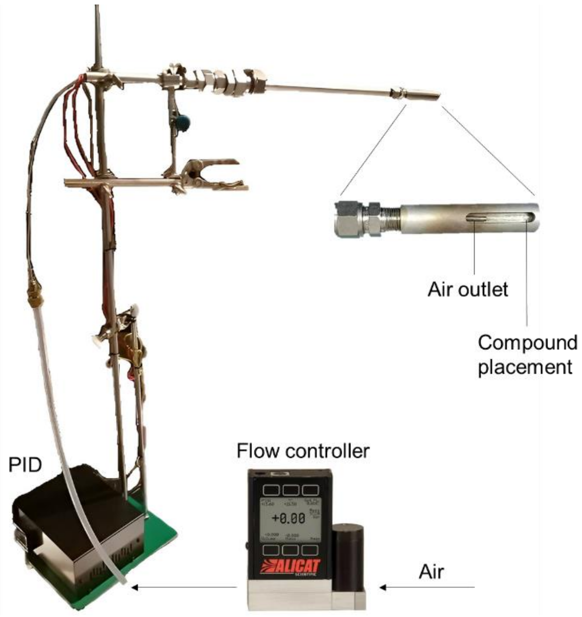

Figure S1. Custom made vaporizer.

The compound is placed on the stainless steel tip located at the top end of the device. Its vaporization is succeeded by controlling the temperature through a PID system. To aid the vaporization and the dispersion of the organic vapors inside the chamber, purified air passes through the devise at a rate of 200 cm^3 min^{-1}, regulated by a flow controller.

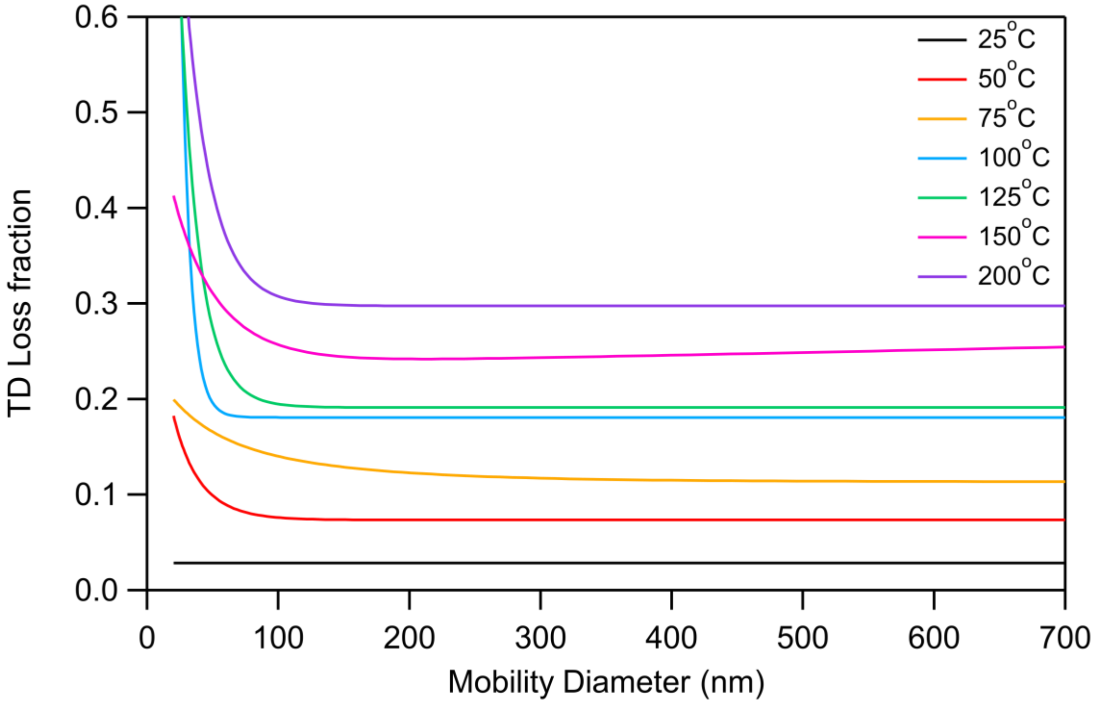

Figure S2. Particle loss fraction in the TD as a function of the mobility diameter for different TD temperatures.

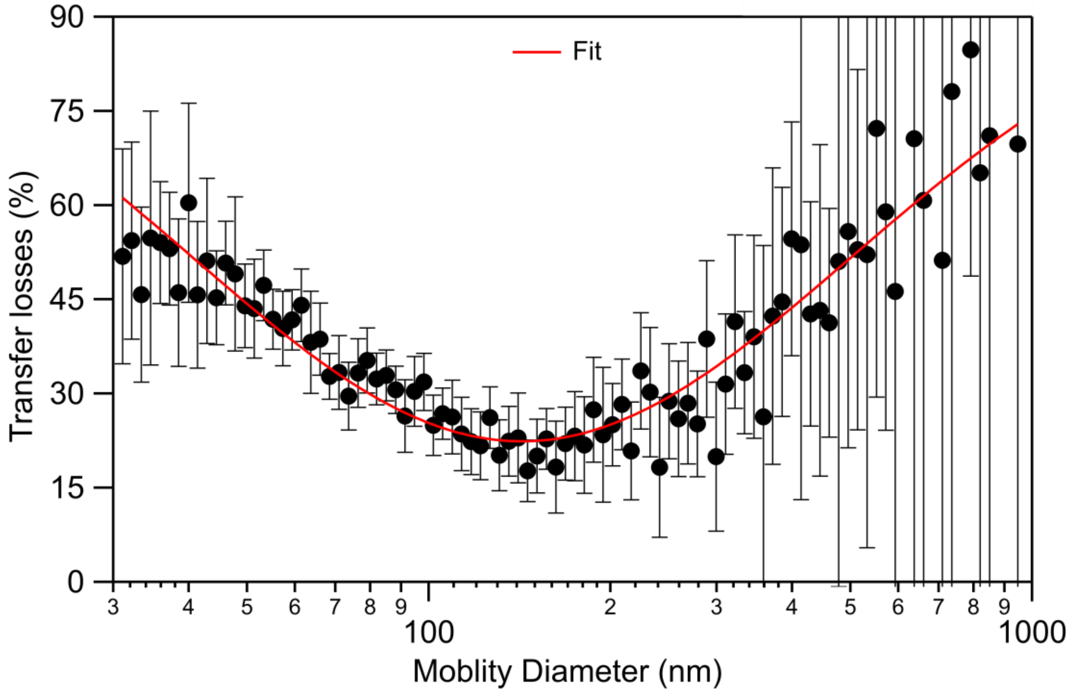

Figure S3. Particle losses as a function of mobility diameter during the transfer of aerosol from the main to the dilution chamber.

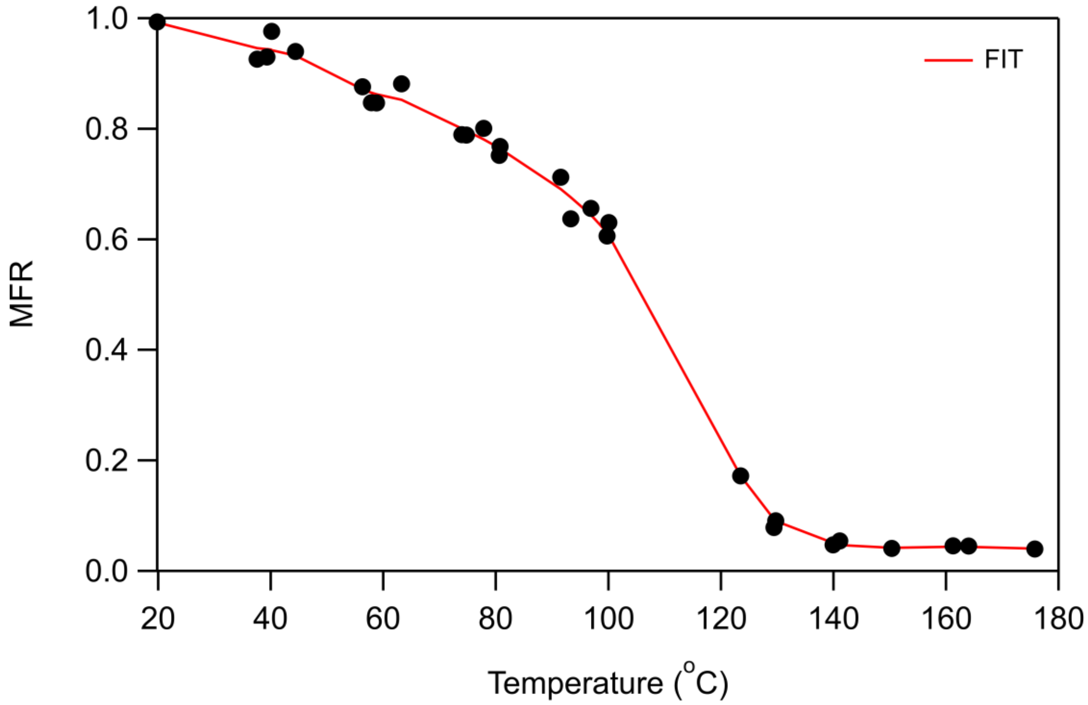

Figure S4. Thermogram of the ammonium sulfate particles.

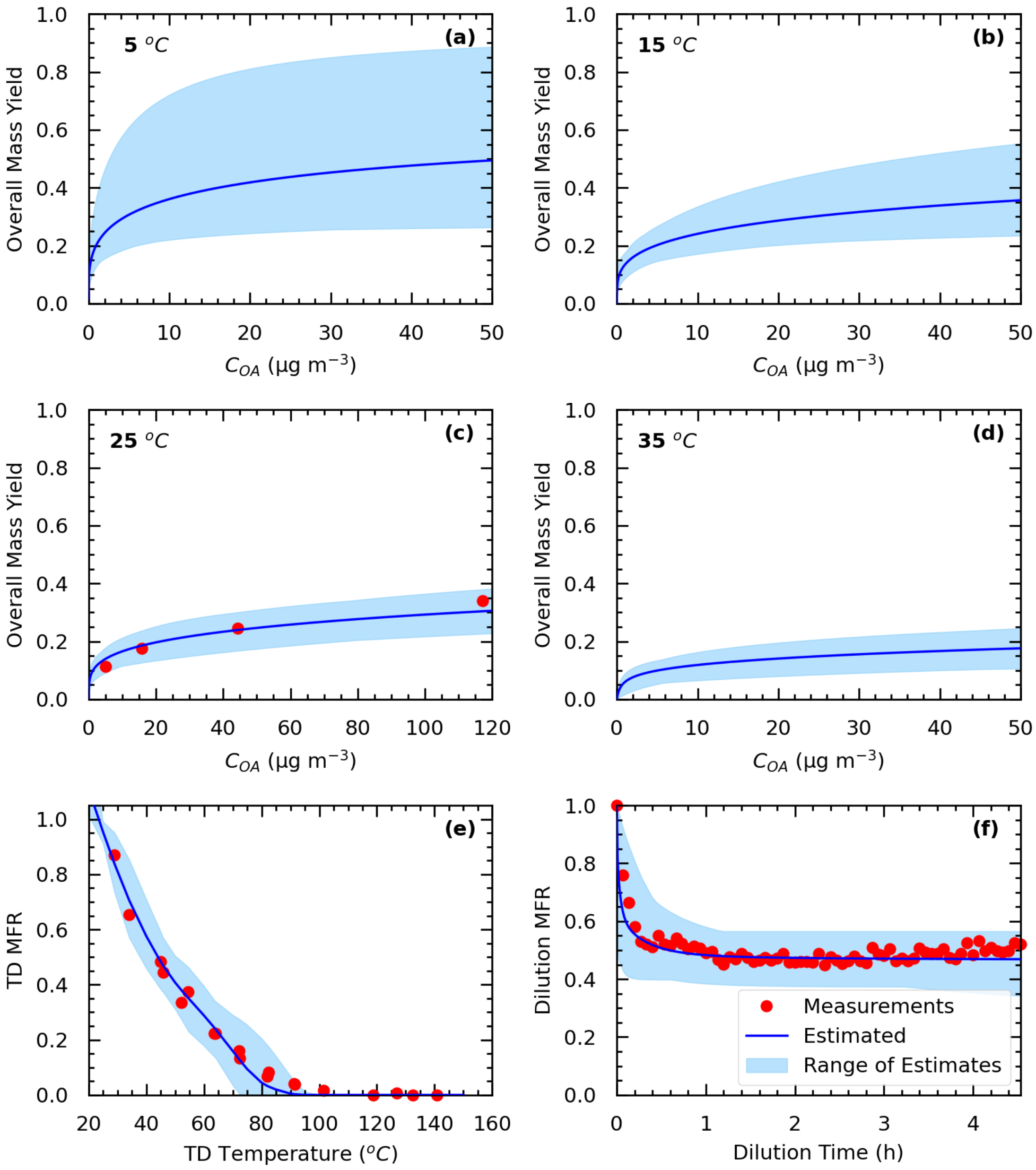

Figure S5. Measurements of amylcyclohexane, estimated (blue line) yields at (a) 5°C (b) 15°C (c) 25°C and (d) 35°C, (e) thermogram and (f) areogram. The blue area shows the range of good solutions. The measurements are corrected for particle wall losses.

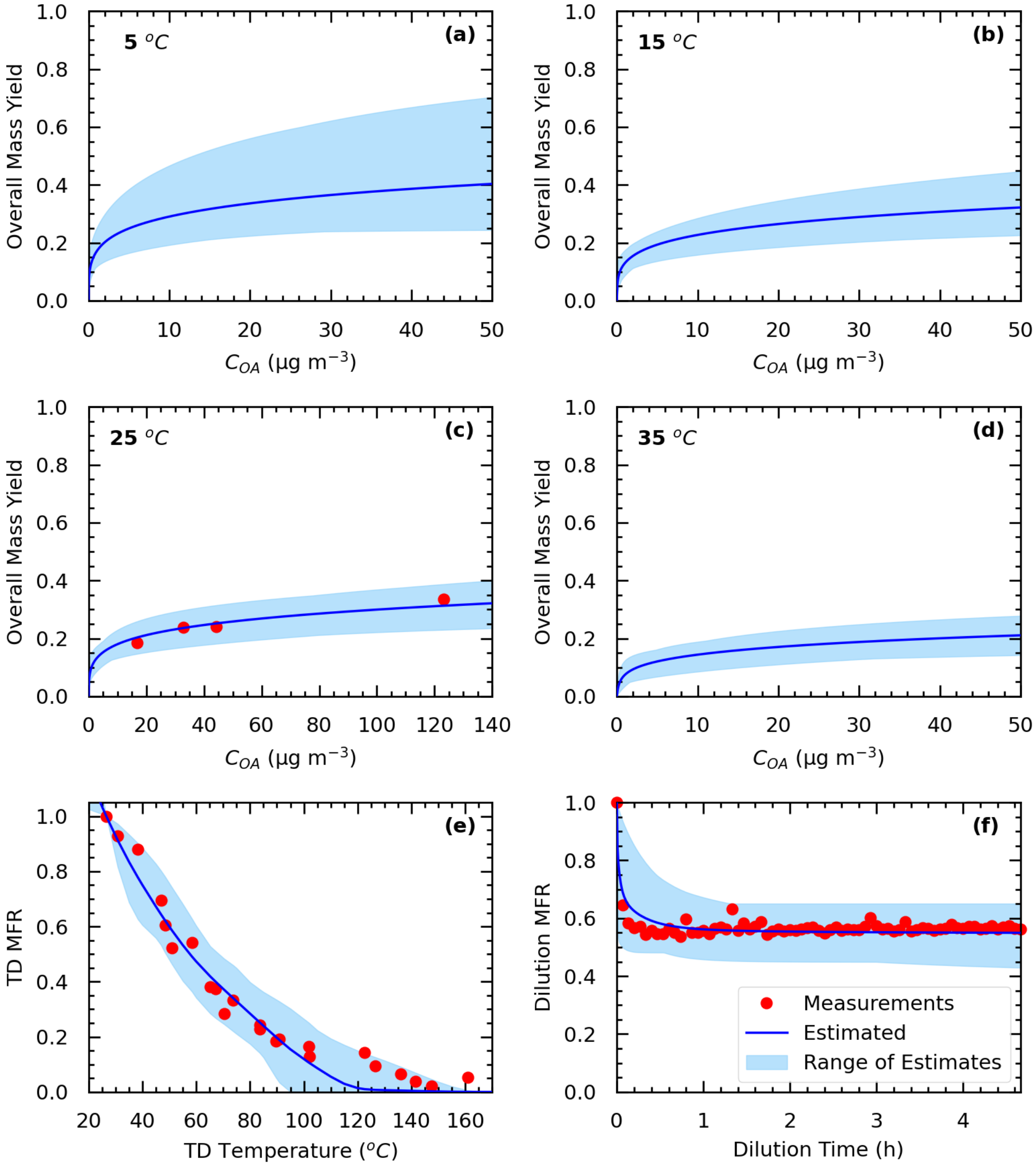

Figure S6. Measurements of hexylcyclohexane, estimated (blue line) yields at (a) 5°C (b) 15°C (c) 25°C and (d) 35°C, (e) thermogram and (f) areogram. The blue area shows the range of good solutions. The measurements are corrected for particle wall losses.

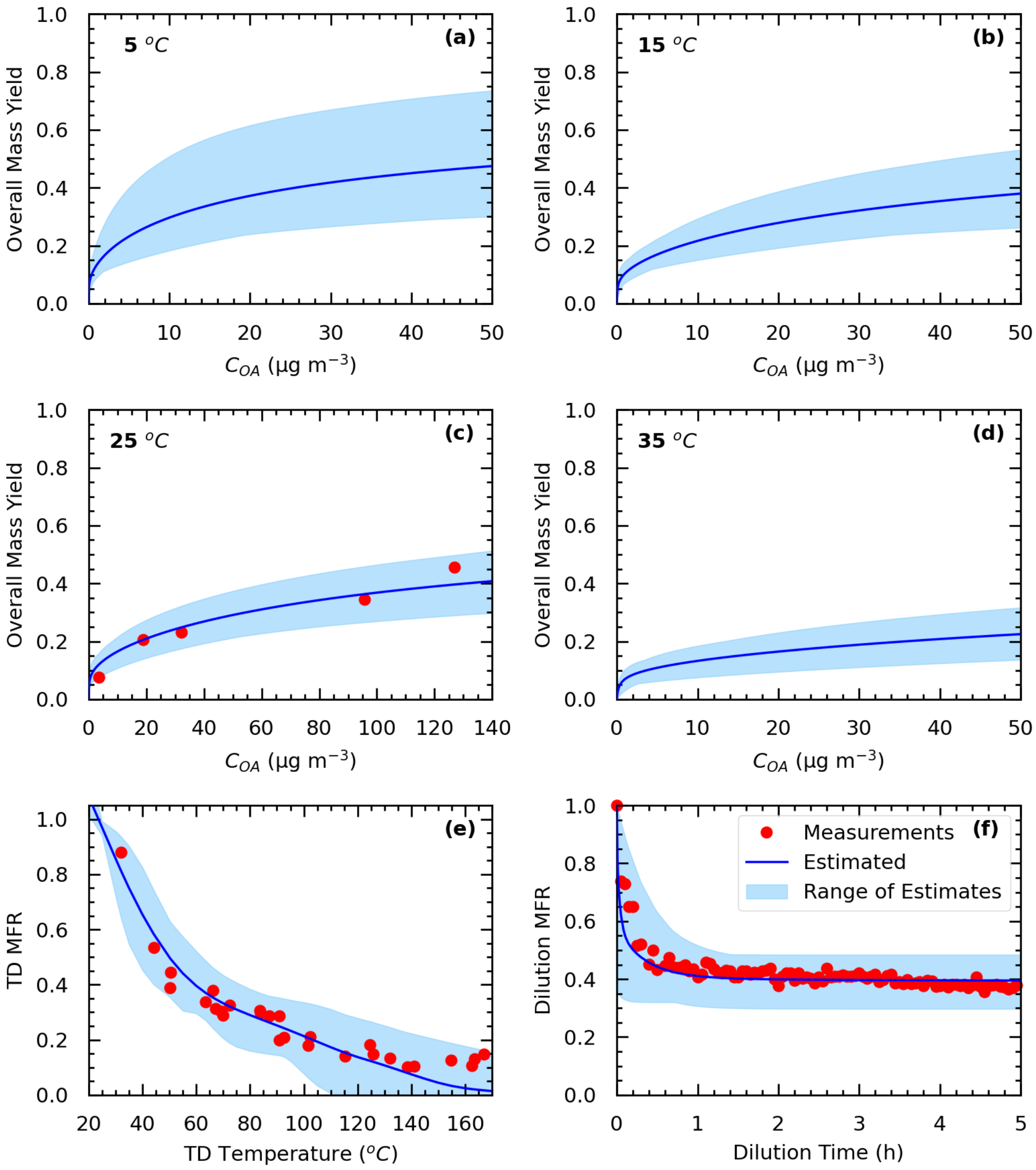

Figure S7. Measurements of nonylcyclohexane, estimated (blue line) yields at (a) 5°C (b) 15°C (c) 25°C and (d) 35°C, (e) thermogram and (f) areogram. The blue area shows the range of good solutions. The measurements are corrected for particle wall losses.

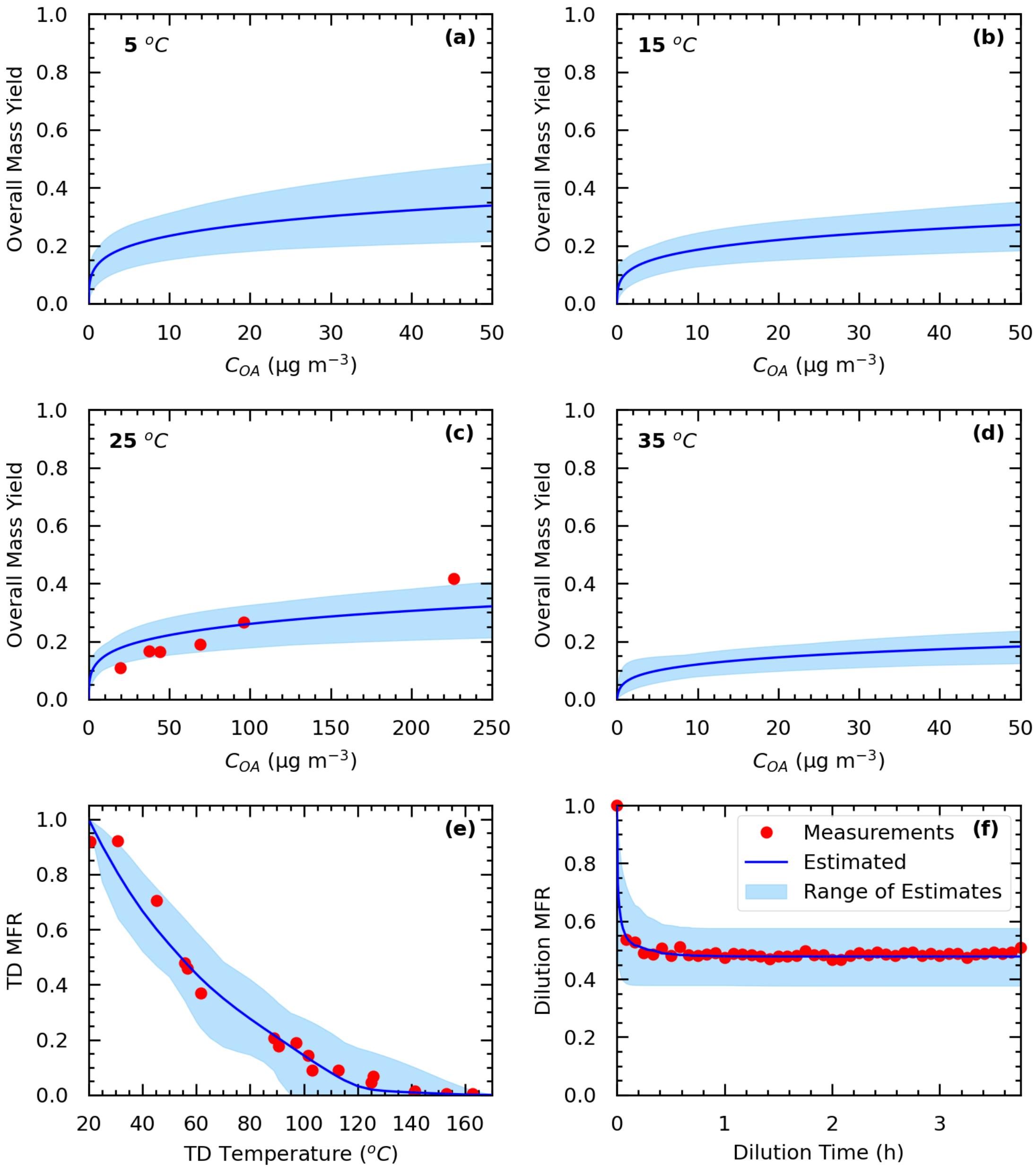

Figure S8. Measurements of decylcyclohexane, estimated (blue line) yields at (a) 5°C (b) 15°C (c) 25°C and (d) 35°C, (e) thermogram and (f) areogram. The blue area shows the range of good solutions. The measurements are corrected for particle wall losses.

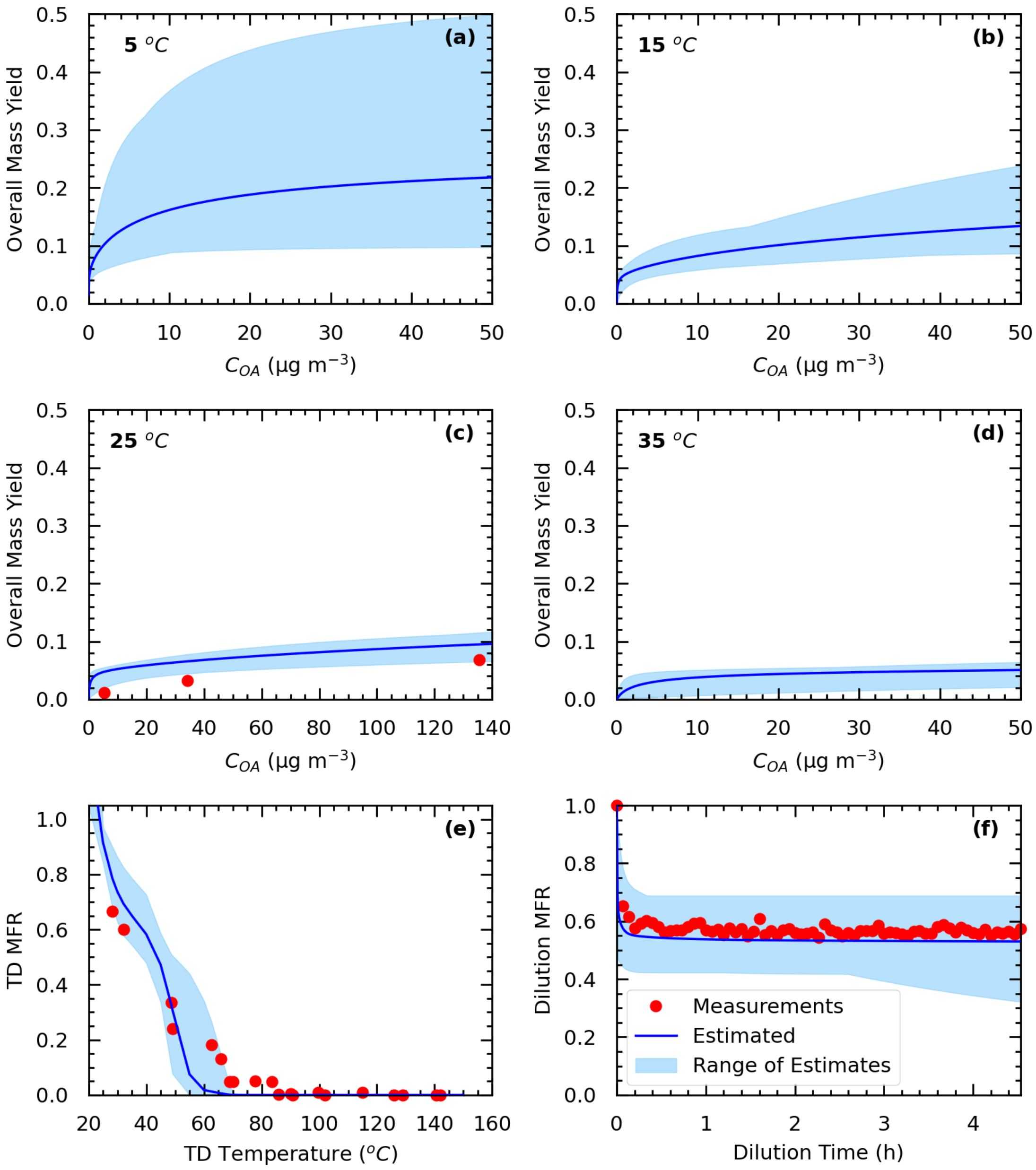

Figure S9. Measurements of 1,3,5-trimethylbenzene, estimated (blue line) yields at (a) 5°C (b) 15°C (c) 25°C and (d) 35°C, (e) thermogram and (f) areogram. The blue area shows the range of good solutions. The measurements are corrected for particle wall losses.

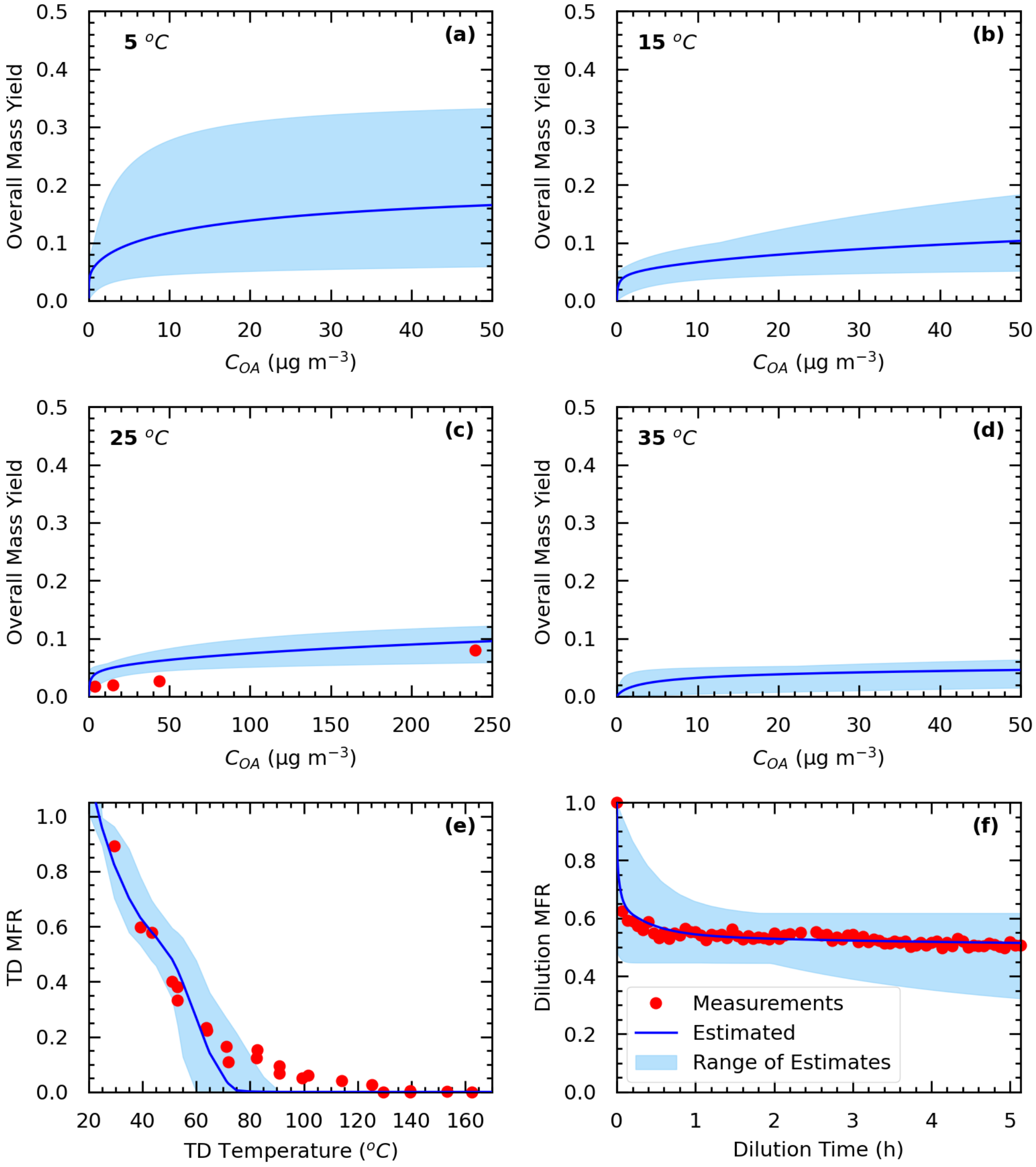

Figure S10. Measurements of 1,3,5-triethylbenzene, estimated (blue line) yields at (a) 5°C (b) 15°C (c) 25°C and (d) 35°C, (e) thermogram and (f) areogram. The blue area shows the range of good solutions. The measurements are corrected for particle wall losses.

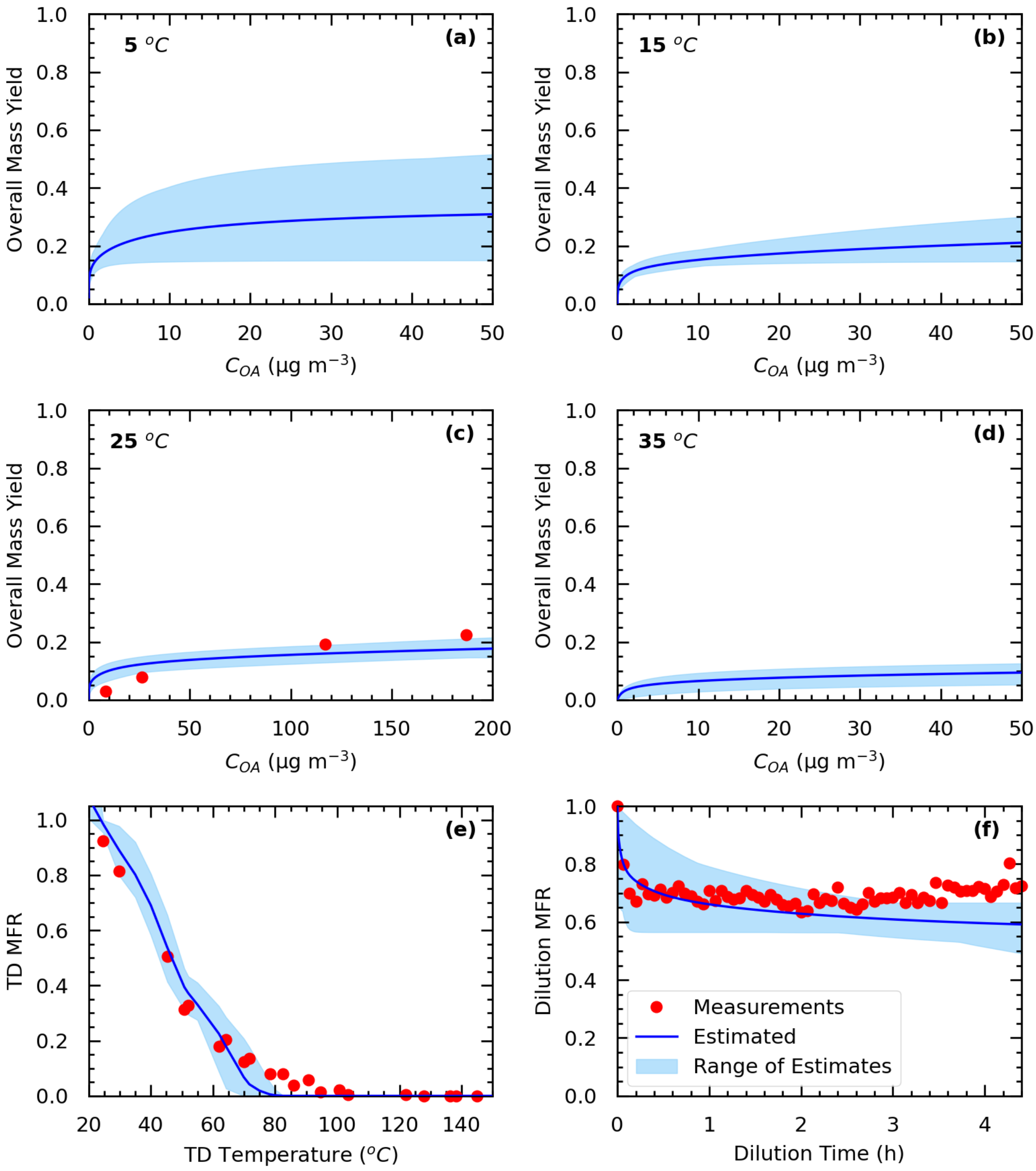

Figure S11. Measurements of 1,3,5-tri-tert-butylbenzene, estimated (blue line) yields at (a) 5°C (b) 15°C (c) 25°C and (d) 35°C, (e) thermogram and (f) areogram. The blue area shows the range of good solutions. The measurements are corrected for particle wall losses.

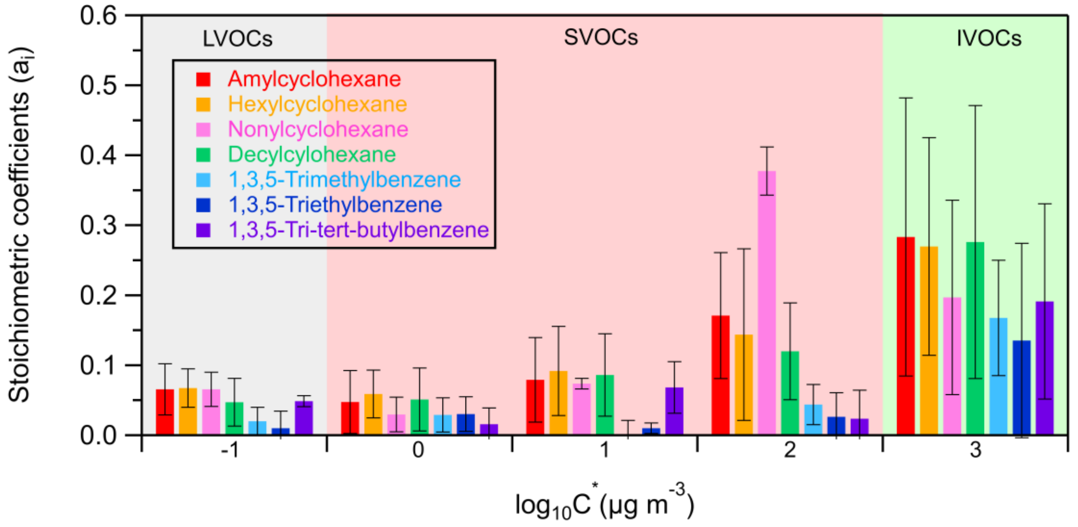

Figure S12. Estimated volatility distributions of the oxidation products of each precursor. In the 1D-VBS the IVOCs are in the green shaded area, SVOCs in pink shaded area and LVOCs in the grey shaded area. The error bars represent the uncertainty in the estimated values.

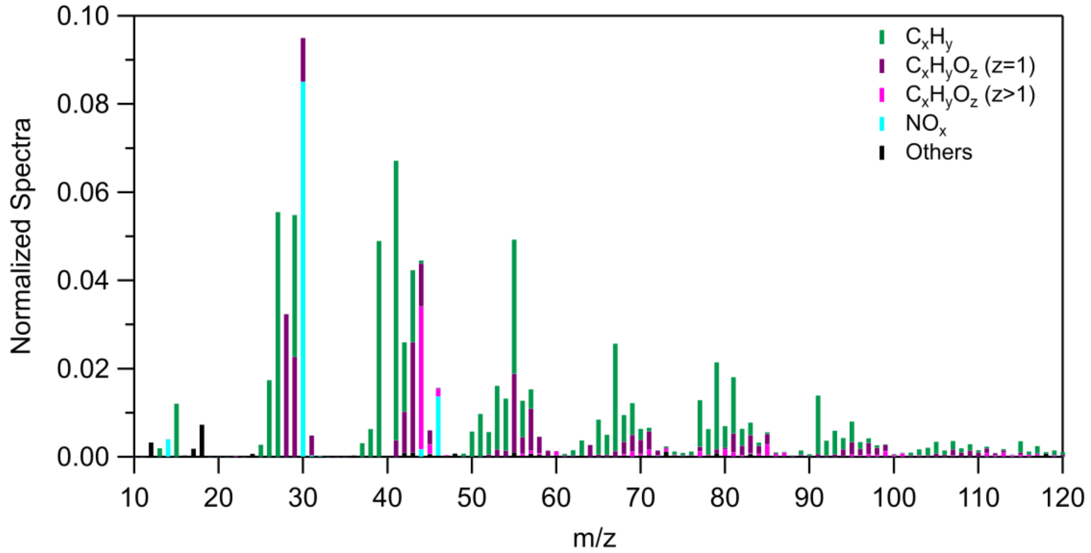

Figure S13. Average normalized HR AMS spectrum of the SOA formed from the oxidation of nonylcyclohexane (NC2-NC5). Water is excluded.

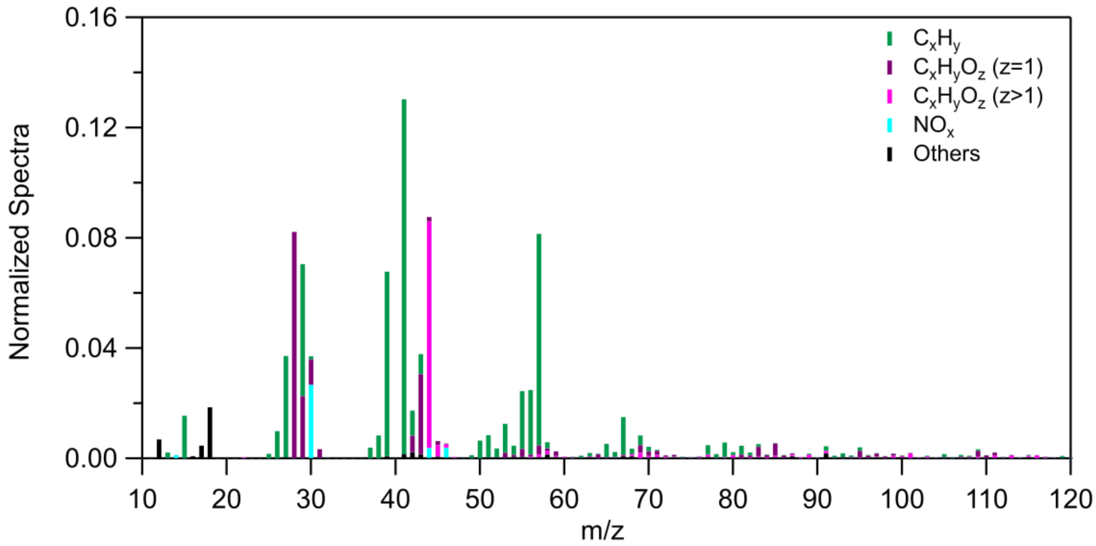

Figure S14. Average normalized HR AMS spectrum of the SOA formed from the oxidation of 1,3,5-tri-tert-butylbenzene (TTB2-TTB4). Water is excluded.

References

1. Nannoolal, Y., Rarey, J., Ramjugernath, D., and Cordes, W. 2004. Estimation of pure component properties: Part 1. Estimation of the normal boiling point of non-electrolyte organic compounds via group contributions and group interactions. *Fluid Phase Equilibria*, *226*, 45–63. https://doi.org/10.1016/j.fluid.2004.09.001
2. Nannoolal, Y., Rarey, J., and Ramjugernath, D. 2008. Estimation of pure component properties. Part 3. Estimation of the vapor pressure of non-electrolyte organic compounds via group contribution and group interactions. *Fluid Phase Equilibria*, *269*, 117–133. https://doi.org/10.1016/j.fluid.2008.04.020
